# Supplementary material for: A multidimensional selective landscape drives adaptive divergence between and within closely related Phlox species
Source: Nat Commun. 2024 May 31;15:4661. doi: 10.1038/s41467-024-49075-6 (PMC11143288; doi:10.1038/s41467-024-49075-6)
Supplement: Supplementary file 1 — Supplementary Information [file 41467_2024_49075_MOESM1_ESM.pdf]

Figure S1: Predicted occurrence of *Phlox amoena* subsp. *amoena* (A), and *Phlox pilosa* subsp. *pilosa* (B) inferred from Maxent ecological niche models. Points indicated all occurrences used to build models. Populations that are also represented in experimental gardens are highlighted in white. A principal component analysis of all environmental data used for niche modeling indicates the two species differ along PC1 and not PC2. Histograms represent the frequency of differences in median value on PC1 (C), median value on PC2 (D), breadth (5th to 95th percentile) on PC1 (E), breadth on PC2 for 1,000 resampled replicates (F). Red lines represent the observed differences in our dataset, and p-values are calculated from a two-sided test comparing observed differences to the differences calculated in 1,000 resampled replicates. Supplemental Table 1 indicates environmental data used to generate niche models and supplemental Table 2 indicates GPS points and environmental variables of known occurrences.

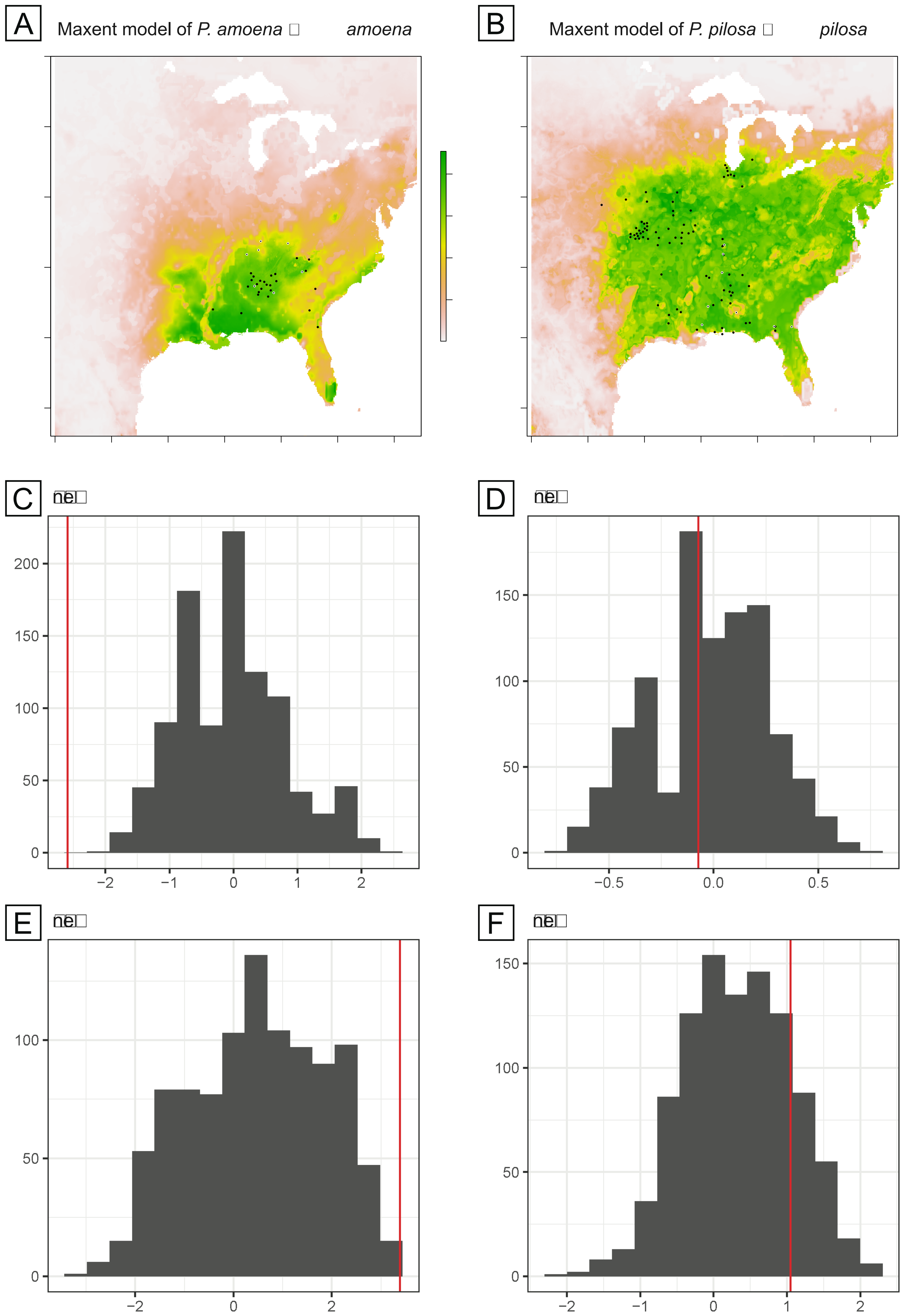

A
Maxent model of P. amoena □ amoena
B
Maxent model of P. pilosa □ pilosa
C
D
E
F
200
150
100
50
0
−2
−1
0
1
2
−0.5
0.0
0.5

Figure S2: Summary of leaf traits from plants grown in greenhouse (n=29 individuals) and in the field (n=72) in box and whisker plot with median indicated by dark bar, lower and upper quartile in box and whiskers indicating spread of data and outliers shown as open circles. All plants were sourced from the same population (#729). Traits include leaf length (A), leaf width (B), leaf area (C), leaf length:width ratio (D), and specific leaf area (E). Amoena individuals are summarized in red and pilosa in blue with field measurements in darker shades and greenhouse measurements in lighter shades.

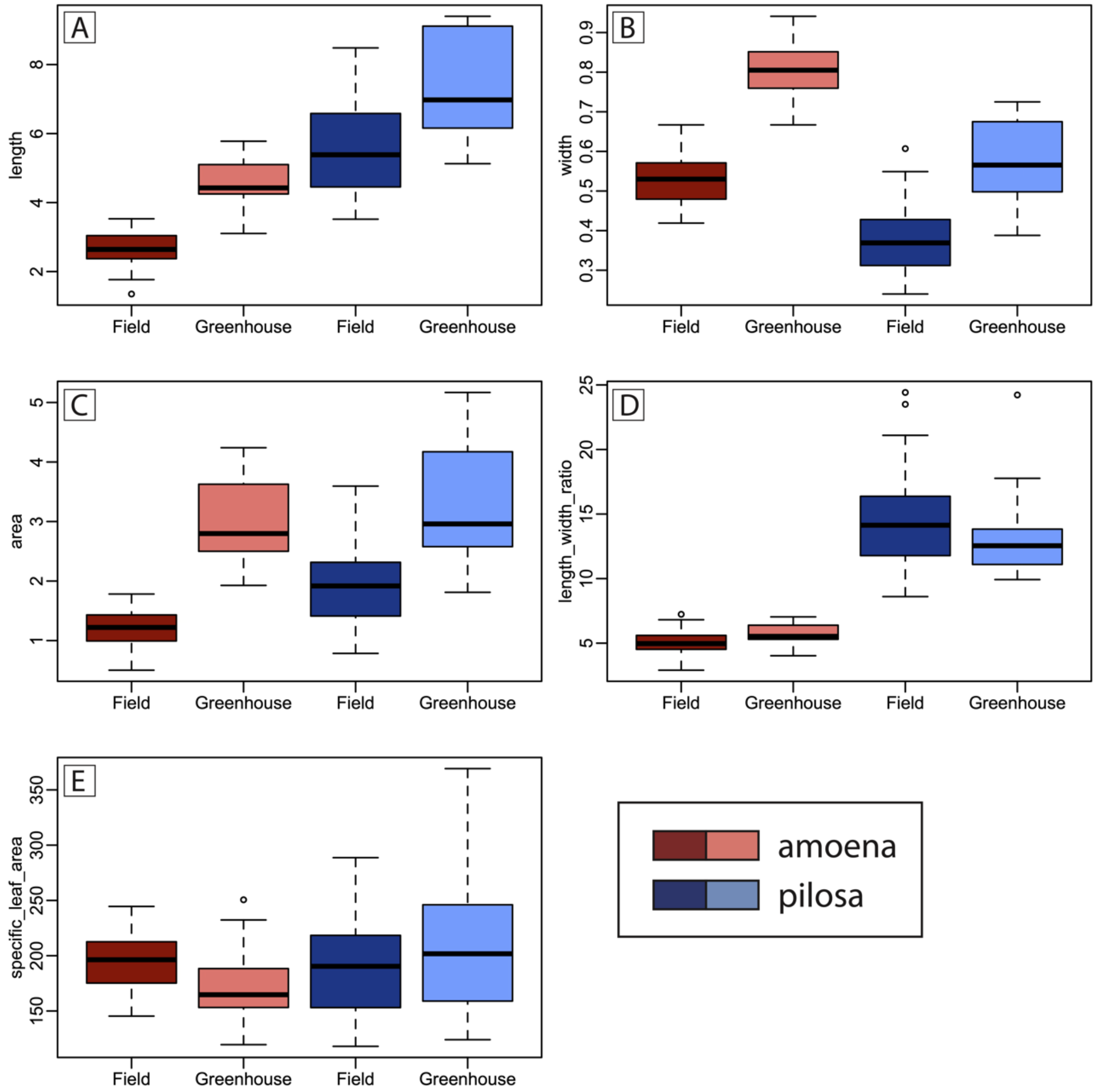

Table S1 Environmental Variables

variable name	variable description	loading PC1	loading PC2	data source
bioclim 1	Mean annual temperature	0.356	0	Worldclim; https://www.worldclim.org/data/index.html
bioclim 2	Mean diurnal temperature range	0.233	0.306	Worldclim; https://www.worldclim.org/data/index.html
bioclim 8	Mean temperature of the wettest quarter	-0.192	-0.274	Worldclim; https://www.worldclim.org/data/index.html
bioclim 12	Annual precipitation	0.387	0	Worldclim; https://www.worldclim.org/data/index.html
Topsoil Sand Fraction	Proportion of topsoil made up by sand (percent weight)	0.311	-0.446	Unified North American Soil Map; https://daac.ornl.gov/NACP/guides/NACP MsTMIP Unified NA Soil Map.html
Topsoil Clay Fraction	Proportion of topsoil made up by clay (percent weight)	-0.241	0.54	Unified North American Soil Map; https://daac.ornl.gov/NACP/guides/NACP MsTMIP Unified NA Soil Map.html
Topsoil Organic Carbon	Proportion of topsoil made up by organic carbon (percent weight)	-0.305	0.133	Unified North American Soil Map; https://daac.ornl.gov/NACP/guides/NACP MsTMIP Unified NA Soil Map.html
Topsoil pH	pH of topsoil	-0.359	0	Unified North American Soil Map; https://daac.ornl.gov/NACP/guides/NACP MsTMIP Unified NA Soil Map.html
Ca	Soil calcium concentration	-0.294	-0.379	USGS Geochemical and Mineralogical Maps; http://pubs.usgs.gov/sir/2017/5118/sir20175118_geo.php
Fe	Soil iron concentration	-0.302	0.201	USGS Geochemical and Mineralogical Maps; http://pubs.usgs.gov/sir/2017/5118/sir20175118_geo.php
Mg	Soil magnesium concentration	-0.279	-0.337	USGS Geochemical and Mineralogical Maps; http://pubs.usgs.gov/sir/2017/5118/sir20175118_geo.php

Table S2 Niche Modeling Points

source	population name	taxon	longitude	latitude	bioclim 1	bioclim 2	bioclim 8	bioclim 12	Topsoil Sand Fraction	Topsoil Clay Fraction	Topsoil Organic Carbon	Topsoil pH	Ca	Fe	Mg
BGS	1701	amoena	-83.1446	34.6822	152	132	73	1468	60	20	0.3163	5.3	0	255	54
BGS	1715	amoena	-85.6546	33.1906	158	138	78	1475	48	25	0.4239	5.25	54	255	0
BGS	1716	amoena	-87.3555	33.6699	160	138	77	1475	65	19	0.705	5.3	0	56	54
BGS	1724	amoena	-86.8048	36.8550	136	126	135	1292	15	35	1.74	5.9	56	210	56
BGS	1726	amoena	-88.0365	35.9193	142	139	142	1377	26	19	0.6128	4.6	0	0	54
BGS	1727	amoena	-87.0001	36.2315	142	136	140	1266	26	19	0.6128	4.6	56	0	56
BGS	1733	amoena	-84.3987	36.7074	125	133	204	1326	43	17	1.595	5	56	54	54
GBIF	1929688596	amoena	-87.0368	33.0596	170	131	91	1460	65	19	0.705	5.3	0	145	54
GBIF	1828869271	amoena	-82.8142	34.7508	154	129	73	1454	54	40	0.2911	5.35	0	255	54
GBIF	1457768129	amoena	-81.7590	30.7545	199	121	267	1305	97	2	0.7932	4.6	0	0	0
GBIF	699265946	amoena	-83.6097	35.6545	112	133	30	1524	44	16	0	5	210	145	0
GBIF	699265945	amoena	-82.5333	35.5581	128	134	219	1155	34	32	0.9106	5.3	255	255	210
GBIF	699265942	amoena	-81.9747	33.4708	171	140	93	1151	93	6	0.435	5.3	0	54	0
GBIF	217075589	amoena	-85.4252	34.0197	149	134	66	1455	28	20	0.87	5.25	54	0	54
GBIF	217075580	amoena	-86.8517	34.0467	155	134	71	1453	68	11	0.725	4.6	54	0	54
GBIF	122983582	amoena	-82.4917	31.9408	187	138	265	1162	76	8	0.435	5.3	0	0	0
GBIF	122241005	amoena	-87.6136	34.0928	152	138	152	1511	69	15	0.4239	5.25	0	54	0
GBIF	56627570	amoena	-86.4939	33.9694	155	132	71	1449	68	11	0.725	4.6	54	0	54
GBIF	56627299	amoena	-87.1392	33.8072	160	137	77	1447	67	16	0.4239	5.25	0	54	54
GBIF	56627297	amoena	-85.8803	34.4175	145	130	61	1464	68	11	0.725	4.6	54	56	54
GBIF	56626923	amoena	-85.9178	33.3636	155	135	75	1477	60	23	0.3286	5	0	145	54
GBIF	56626785	amoena	-85.9614	33.7239	160	135	78	1384	26	19	0.6128	4.6	0	145	54
GBIF	56626781	amoena	-86.7506	33.4781	163	126	81	1428	65	19	0.705	5.3	0	0	54
GBIF	56626778	amoena	-87.9364	33.6347	161	141	116	1490	51	26	0.435	4.6	0	56	54
GBIF	56626773	amoena	-85.5197	34.5258	139	130	54	1500	68	11	0.725	4.6	54	56	54
GBIF	56626771	amoena	-87.6667	34.5833	154	135	153	1421	9	45	0.733	4.6	0	54	0
GBIF	56626769	amoena	-86.1514	32.9222	165	136	87	1465	60	20	0.3163	5.3	0	255	54
GBIF	56626757	amoena	-87.4395	34.3483	151	132	151	1491	69	15	0.4239	5.25	0	56	54
GBIF	56626752	amoena	-86.9767	33.2397	167	132	87	1466	65	19	0.705	5.3	0	0	54
GBIF	56626749	amoena	-87.3861	33.6722	161	138	77	1478	65	19	0.705	5.3	0	56	54
GBIF	56626648	amoena	-86.2828	33.7661	159	134	77	1415	68	11	0.725	4.6	0	56	54
SERNEC	6189178	amoena	-88.5239	31.7440	178	142	105	1515	70	8	0.725	5	0	0	0
SERNEC	6349345	amoena	-91.0317	32.0089	179	131	104	1431	18	57	1.0024	6.8	56	210	0
BGS	1704	pilosa	-81.9733	30.7763	198	131	269	1318	97	2	0.7932	4.6	0	0	0
BGS	1708	pilosa	-83.5291	30.8033	194	132	267	1305	76	8	0.435	5.3	0	0	0

Table S2 Niche Modeling Points

source	population name	taxon	longitude	latitude	bioclim 1	bioclim 2	bioclim 8	bioclim 12	Topsoil Sand Fraction	Topsoil Clay Fraction	Topsoil Organic Carbon	Topsoil pH	Ca	Fe	Mg
BGS	1709	pilosa	-83.4258	30.7899	194	131	268	1297	76	8	0.435	5.3	0	0	0
BGS	1710	pilosa	-83.4326	30.4955	196	131	268	1356	95	4	0.435	5.3	0	0	0
BGS	1712	pilosa	-89.9061	30.9155	189	129	116	1631	63	17	0.6432	5.42	0	54	0
BGS	1713	pilosa	-89.3791	32.2189	174	135	132	1470	68	9	0.725	5	54	56	56
BGS	1714	pilosa	-86.8710	31.7884	179	136	104	1460	53	25	0.435	4.6	255	210	145
BGS	1718	pilosa	-88.1387	34.6280	156	137	156	1425	66	9	0.725	5	54	56	54
BGS	1722	pilosa	-88.1052	36.4995	142	126	143	1333	11	21	1.45	5	0	0	56
BGS	1723	pilosa	-87.9385	36.5969	141	124	141	1308	10	23	1.74	5.76	0	56	56
BGS	1725	pilosa	-87.9921	35.8922	142	140	142	1383	25	22	1.74	5.5	0	0	54
BGS	1728	pilosa	-88.0433	35.9440	143	138	143	1371	26	19	0.6128	4.6	0	0	54
BGS	1730	pilosa	-88.0089	36.5661	143	125	143	1312	11	21	1.45	5	0	0	56
GBIF	1563145207	pilosa	-88.7875	30.3969	197	100	268	1601	59	11	0.435	5	56	56	56
GBIF	1269646240	pilosa	-92.1160	32.0160	181	130	88	1440	57	21	0.9078	5.14	54	54	54
GBIF	1269646228	pilosa	-92.8330	31.6160	188	129	95	1409	23	48	0.9106	7.5	54	56	56
GBIF	1269646222	pilosa	-91.7830	31.8160	184	126	92	1453	49	28	0.725	5	0	255	210
GBIF	1261001335	pilosa	-90.9072	39.0394	122	127	218	964	7	22	0.725	6.2	0	145	0
GBIF	1260999485	pilosa	-92.1086	40.3250	108	123	221	941	31	37	0.8279	6	0	255	0
GBIF	1260985283	pilosa	-91.5167	38.0272	124	135	176	1031	26	21	0.9687	4.98	54	145	0
GBIF	1260977818	pilosa	-91.7036	36.7022	131	142	131	1133	31	11	0.559	5.63	54	56	54
GBIF	1260944387	pilosa	-92.4664	39.0108	122	117	220	992	28	43	0.2392	5.5	0	0	0
GBIF	1258168527	pilosa	-93.9864	37.4992	133	128	182	1092	13	16	1.2912	6.37	56	0	54
GBIF	1258052296	pilosa	-92.4511	38.6964	129	122	181	977	3	25	0.6464	6.15	0	210	0
GBIF	1257984306	pilosa	-90.5186	37.9865	124	133	124	1058	6	19	0.8735	6.83	255	210	255
GBIF	1257795317	pilosa	-94.7783	39.6736	117	123	220	929	7	31	1.139	6.5	145	255	210
GBIF	1257724248	pilosa	-94.8722	40.3461	103	129	209	883	18	32	2.03	6.2	145	255	210
GBIF	1257724240	pilosa	-92.4947	39.6581	115	119	216	1014	28	43	0.2392	5.5	0	0	56
GBIF	1257723853	pilosa	-91.7358	37.1003	131	134	131	1099	20	13	0.7886	4.84	54	0	56
GBIF	1257723640	pilosa	-92.2589	37.1108	127	128	175	1097	23	12	0.7616	5.13	54	56	54
GBIF	1257689060	pilosa	-91.6736	37.4589	129	133	130	1075	22	11	1.2137	4.61	54	56	56
GBIF	1257688477	pilosa	-93.8231	36.5761	135	134	183	1118	13	10	0.8495	5.67	56	0	54
GBIF	1228496571	pilosa	-87.6501	41.8500	100	99	223	921	35	24	1.68	6.2	255	255	255
GBIF	1228447146	pilosa	-87.0733	41.5088	95	109	215	978	26	27	1.16	6.2	0	0	0
GBIF	1228418322	pilosa	-87.6102	41.5441	99	106	221	945	18	38	3.19	6.5	210	210	255
GBIF	1228401864	pilosa	-87.7228	42.0723	95	98	217	906	35	24	1.68	6.2	255	255	255
GBIF	1228555232	pilosa	-87.9667	41.4333	96	112	198	945	20	26	2.61	6.5	255	210	255

Table S2 Niche Modeling Points

source	population name	taxon	longitude	latitude	bioclim 1	bioclim 2	bioclim 8	bioclim 12	Topsoil Sand Fraction	Topsoil Clay Fraction	Topsoil Organic Carbon	Topsoil pH	Ca	Fe	Mg
GBIF	1228183408	pilosa	-87.3662	41.5931	99	107	219	939	89	5	0.3286	5.9	210	145	255
GBIF	1137119013	pilosa	-93.5002	34.4956	149	136	148	1448	36	25	0.5815	5.23	54	56	54
GBIF	1137119006	pilosa	-98.7883	39.4389	117	152	223	628	42	21	0.4903	7.9	255	145	210
GBIF	1137119000	pilosa	-94.6843	37.0237	142	126	190	1086	22	14	0.87	5.59	56	145	56
GBIF	699266035	pilosa	-86.3567	40.7544	99	117	199	954	54	16	0.6176	6.16	210	56	0
GBIF	217076988	pilosa	-87.3914	33.7078	161	138	77	1478	65	19	0.705	5.3	0	56	54
GBIF	217076879	pilosa	-87.5008	34.4889	155	134	154	1438	69	15	0.4239	5.25	0	54	54
GBIF	217076457	pilosa	-88.1786	32.6642	172	135	130	1426	66	9	0.725	5	54	56	54
GBIF	217076456	pilosa	-86.3314	34.4550	154	130	68	1415	68	11	0.725	4.6	56	145	54
GBIF	217076455	pilosa	-87.3989	32.4436	177	131	100	1370	14	62	2.9	5.5	0	255	56
GBIF	177002340	pilosa	-95.2418	38.2326	131	124	227	1015	10	21	1.16	6.1	0	255	0
GBIF	177002334	pilosa	-95.6825	37.3979	140	132	233	998	27	18	1.16	5.6	0	255	145
GBIF	177002332	pilosa	-95.7339	37.7925	135	128	228	999	15	34	1.3702	6.08	210	255	145
GBIF	177002327	pilosa	-96.2289	37.3732	139	136	231	946	25	32	1.015	6.28	0	255	145
GBIF	177002326	pilosa	-96.6424	39.8201	114	133	221	801	34	34	2.03	6.5	210	210	210
GBIF	177002323	pilosa	-95.1207	37.1427	140	127	191	1059	19	36	1.45	6.5	0	255	0
GBIF	177002317	pilosa	-95.0250	38.1042	132	124	185	1035	10	21	1.16	6.1	0	255	0
GBIF	177002313	pilosa	-94.9760	37.7631	136	124	188	1055	27	18	1.16	5.6	0	255	0
GBIF	177002310	pilosa	-94.7888	37.6485	136	125	188	1080	27	18	1.16	5.6	56	255	0
GBIF	177002307	pilosa	-95.5894	37.2309	141	132	191	1020	19	36	1.16	6.12	0	255	0
GBIF	177002305	pilosa	-95.8023	37.0336	142	133	192	997	27	18	1.16	5.6	56	255	0
GBIF	177002298	pilosa	-94.7767	38.1428	130	126	184	1041	10	21	1.16	6.1	0	255	210
GBIF	177002290	pilosa	-95.4798	37.0464	141	132	192	1044	19	36	1.45	6.5	0	255	0
GBIF	177002289	pilosa	-95.8916	37.2873	141	135	191	981	10	21	1.16	6.1	145	255	145
GBIF	177002287	pilosa	-94.7637	37.9231	134	125	186	1058	27	18	1.16	5.6	0	255	0
GBIF	177002277	pilosa	-95.1276	37.5334	137	124	190	1043	27	18	1.16	5.6	0	255	0
GBIF	177002269	pilosa	-95.4628	37.6128	136	126	230	997	15	34	1.3702	6.08	145	255	0
GBIF	177002261	pilosa	-95.3426	37.4172	136	125	188	1030	15	34	1.3702	6.08	0	255	0
GBIF	177002255	pilosa	-96.2454	37.1501	142	137	191	954	25	32	1.015	6.28	56	56	56
GBIF	177001663	pilosa	-85.4640	42.6530	86	112	195	901	69	7	1.015	6.2	56	54	56
GBIF	56627981	pilosa	-87.3431	32.9972	170	127	92	1436	68	9	0.725	5	0	54	0
GBIF	56626934	pilosa	-87.4703	33.2703	168	132	86	1438	68	9	0.725	5	0	56	54
GBIF	56626932	pilosa	-87.2108	33.2067	167	129	86	1469	65	19	0.705	5.3	0	56	54
GBIF	56626928	pilosa	-87.9736	33.3711	166	135	121	1467	68	9	0.725	5	0	56	54
GBIF	56626921	pilosa	-85.7575	33.6697	152	134	70	1454	26	19	0.6128	4.6	0	255	54

Table S2 Niche Modeling Points

**source**	**population name**	**taxon**	**longitude**	**latitude**	**bioclim 1**	**bioclim 2**	**bioclim 8**	**bioclim 12**	**Topsoil Sand Fraction**	**Topsoil Clay Fraction**	**Topsoil Organic Carbon**	**Topsoil pH**	**Ca**	**Fe**	**Mg**
GBIF	56626908	pilosa	-86.0306	31.0331	189	131	266	1494	67	11	0.725	5.3	0	0	0
GBIF	56626906	pilosa	-85.6867	31.0592	189	127	266	1410	67	14	0.435	5.3	0	54	0
GBIF	56626904	pilosa	-88.1333	30.6833	193	112	267	1597	67	10	0.725	4.6	0	54	0
SERNEC	10638097	pilosa	-93.7244	37.0931	129	124	177	1103	13	16	1.2912	6.37	56	210	54
SERNEC	10638102	pilosa	-92.7753	36.7339	137	135	184	1093	14	47	0.8368	6.02	54	54	54
SERNEC	10780686	pilosa	-90.3550	30.5628	191	130	268	1614	15	16	0.7889	5.3	0	54	0
SERNEC	10814730	pilosa	-91.1003	37.4175	131	138	132	1104	56	12	1.16	5.3	54	56	54
SERNEC	10857246	pilosa	-92.8778	37.7403	132	129	182	1067	25	9	0.7266	5	56	210	0
SERNEC	10890118	pilosa	-90.6825	37.5417	127	135	127	1128	16	17	1.6952	4.86	0	0	0
SERNEC	4380991	pilosa	-87.8577	42.2630	88	101	208	888	18	28	1.1895	6.32	210	255	255
SERNEC	5672445	pilosa	-93.8083	32.3072	182	127	222	1230	51	24	0.435	5.07	54	56	0
SERNEC	5672508	pilosa	-92.8750	31.0208	189	127	111	1509	17	14	1.2936	5.45	54	54	54
SERNEC	5906054	pilosa	-89.2785	34.2631	161	130	161	1451	66	9	0.725	5	54	0	54
SERNEC	5906056	pilosa	-89.5328	34.3888	158	129	157	1442	66	9	0.725	5	54	0	54
SERNEC	6362538	pilosa	-88.9150	32.3183	174	136	133	1416	40	35	0.435	4.3	0	54	0
BGS	1719	deamii	-87.5229	37.1850	139	123	140	1233	22	22	1.16	5	56	255	0
BGS	1720	deamii	-87.4845	37.1497	140	124	141	1231	14	37	0.7015	5.9	56	255	0
BGS	1721	deamii	-88.1280	36.9404	140	121	141	1284	68	12	0.7195	5	56	145	56
BGS	1731	deamii	-88.0842	36.8338	141	121	142	1291	68	12	0.7195	5	56	145	56
BGS	1732	deamii	-87.4962	37.0925	140	124	140	1239	14	37	0.7015	5.9	56	210	0

Table S3 Population distances and location (by individual)

garden	taxon	pop	genotype	longitude	latitude	geo_dist	gen_dist	env_dist
AMO	pilosa	1718	1718_20	-88.13868	34.62799	206226.31	0.58901	1.1653841
AMO	amoena	1716	1716_09	-87.35549	33.66987	287001.64	0.43994	1.0386371
AMO	pilosa	1713	1713_10	-89.37914	32.21889	497422.69	0.57193	1.3723948
AMO	deamii	1720	1720_03	-87.48447	37.14971	110978.38	0.54881	2.7703831
AMO	amoena	1715	1715_06	-85.65458	33.19064	360196.47	0.3062	1.2329308
AMO	pilosa	1718	1718_05	-88.13868	34.62799	206226.31	0.58901	1.1653841
AMO	deamii	1721	1721_15	-88.12802	36.9404	128027.97	0.59141	1.3118596
AMO	amoena	1716	1716_03	-87.35549	33.66987	287001.64	0.43994	1.0386371
AMO	amoena	1733	1733_11	-84.39869	36.70741	238817.35	0.28783	1.1824762
AMO	amoena	1726	1726_02	-88.03654	35.91927	99521.44	0.33102	0.6676597
AMO	pilosa	1729	1729_12	-88.0375	35.92486	99382.99	0.54341	0.6676597
AMO	pilosa	1718	1718_14	-88.13868	34.62799	206226.31	0.58901	1.1653841
AMO	pilosa	1713	1713_03	-89.37914	32.21889	497422.69	0.57193	1.3723948
AMO	amoena	1701	1701_05	-83.14457	34.68221	389785.01	0.36717	0.6131125
AMO	amoena	1727	1727_07	-87.00008	36.23154	0	0	0
AMO	pilosa	1708	1708_01	-83.52907	30.8033	684636.29	0.65094	1.872317
AMO	deamii	1504	1504_BG15	-87.4961	37.0928	105611.01	0.54881	2.7703831
AMO	pilosa	1704	1704_10	-81.97334	30.77629	765556.29	0.65699	2.7875778
AMO	amoena	1726	1726_19	-88.03654	35.91927	99521.44	0.33102	0.6676597
AMO	amoena	1724	1724_15	-86.80475	36.85496	71563.35	0.17577	3.1715039
AMO	deamii	1719	1719_11	-87.52286	37.18501	115939.77	0.56193	1.7926634
AMO	pilosa	1709	1709_06	-83.42582	30.78985	690513.38	0.65094	1.872317
AMO	amoena	1701	1701_01	-83.14457	34.68221	389785.01	0.36717	0.6131125
AMO	deamii	1505	1505_BG17	-87.4839	37.15035	111024.47	0.54881	2.7703831
AMO	pilosa	1714	1714_14	-86.87098	31.78844	494746.81	0.5798	1.0389322
AMO	pilosa	1714	1714_02	-86.87098	31.78844	494746.81	0.5798	1.0389322
AMO	amoena	1716	1716_11	-87.35549	33.66987	287001.64	0.43994	1.0386371
AMO	pilosa	1723	1723_12	-87.93854	36.59687	93389.65	0.5682	2.02107
AMO	pilosa	1729	1729_06	-88.0375	35.92486	99382.99	0.54341	0.6676597
AMO	deamii	1721	1721_19	-88.12802	36.9404	128027.97	0.59141	1.3118596
AMO	amoena	1715	1715_15	-85.65458	33.19064	360196.47	0.3062	1.2329308
AMO	amoena	1733	1733_03	-84.39869	36.70741	238817.35	0.28783	1.1824762
AMO	pilosa	1729	1729_22	-88.0375	35.92486	99382.99	0.54341	0.6676597
AMO	pilosa	1714	1714_19	-86.87098	31.78844	494746.81	0.5798	1.0389322
AMO	pilosa	1728	1728_17	-88.04331	35.94396	99156.7	0.56851	0.6234453
AMO	amoena	1715	1715_11	-85.65458	33.19064	360196.47	0.3062	1.2329308
AMO	amoena	1727	1727_14	-87.00008	36.23154	0	0	0
AMO	deamii	1720	1720_14	-87.48447	37.14971	110978.38	0.54881	2.7703831
AMO	deamii	1720	1720_09	-87.48447	37.14971	110978.38	0.54881	2.7703831
AMO	deamii	1719	1719_08	-87.52286	37.18501	115939.77	0.56193	1.7926634
AMO	amoena	1716	1716_18	-87.35549	33.66987	287001.64	0.43994	1.0386371
AMO	amoena	1701	1701_18	-83.14457	34.68221	389785.01	0.36717	0.6131125
AMO	pilosa	1723	1723_17	-87.93854	36.59687	93389.65	0.5682	2.02107
AMO	pilosa	1723	1723_19	-87.93854	36.59687	93389.65	0.5682	2.02107
AMO	pilosa	1713	1713_14	-89.37914	32.21889	497422.69	0.57193	1.3723948
AMO	amoena	1729	1729_64	-88.0375	35.92486	99382.99	0.33658	0.6676597
AMO	pilosa	1728	1728_08	-88.04331	35.94396	99156.7	0.56851	0.6234453
AMO	amoena	1724	1724_18	-86.80475	36.85496	71563.35	0.17577	3.1715039
AMO	pilosa	1718	1718_09	-88.13868	34.62799	206226.31	0.58901	1.1653841
AMO	pilosa	1714	1714_04	-86.87098	31.78844	494746.81	0.5798	1.0389322
AMO	deamii	1720	1720_16	-87.48447	37.14971	110978.38	0.54881	2.7703831
AMO	deamii	1719	1719_19	-87.52286	37.18501	115939.77	0.56193	1.7926634

Table S3 Population distances and location (by individual)

garden	taxon	pop	genotype	longitude	latitude	geo_dist	gen_dist	env_dist
AMO	pilosa	1723	1723_07	-87.93854	36.59687	93389.65	0.5682	2.02107
AMO	amoena	1727	1727_02	-87.00008	36.23154	0	0	0
AMO	deamii	1720	1720_20	-87.48447	37.14971	110978.38	0.54881	2.7703831
AMO	pilosa	1729	1729_17	-88.0375	35.92486	99382.99	0.54341	0.6676597
AMO	deamii	1720	1720_03	-87.48447	37.14971	110978.38	0.54881	2.7703831
AMO	pilosa	1728	1728_25	-88.04331	35.94396	99156.7	0.56851	0.6234453
AMO	amoena	1724	1724_08	-86.80475	36.85496	71563.35	0.17577	3.1715039
AMO	deamii	1720	1720_06	-87.48447	37.14971	110978.38	0.54881	2.7703831
AMO	amoena	1726	1726_13	-88.03654	35.91927	99521.44	0.33102	0.6676597
AMO	pilosa	1712	1712_13	-89.90611	30.91547	650175.32	0.59966	1.5227537
AMO	pilosa	1712	1712_03	-89.90611	30.91547	650175.32	0.59966	1.5227537
AMO	amoena	1715	1715_02	-85.65458	33.19064	360196.47	0.3062	1.2329308
AMO	deamii	1719	1719_20	-87.52286	37.18501	115939.77	0.56193	1.7926634
AMO	pilosa	1712	1712_09	-89.90611	30.91547	650175.32	0.59966	1.5227537
AMO	amoena	1727	1727_12	-87.00008	36.23154	0	0	0
AMO	pilosa	1713	1713_07	-89.37914	32.21889	497422.69	0.57193	1.3723948
AMO	amoena	1729	1729_57	-88.0375	35.92486	99382.99	0.33658	0.6676597
AMO	amoena	1724	1724_04	-86.80475	36.85496	71563.35	0.17577	3.1715039
AMO	pilosa	1723	1723_02	-87.93854	36.59687	93389.65	0.5682	2.02107
AMO	amoena	1701	1701_12	-83.14457	34.68221	389785.01	0.36717	0.6131125
AMO	amoena	1729	1729_48	-88.0375	35.92486	99382.99	0.33658	0.6676597
AMO	deamii	1719	1719_01	-87.52286	37.18501	115939.77	0.56193	1.7926634
AMO	amoena	1726	1726_09	-88.03654	35.91927	99521.44	0.33102	0.6676597
AMO	amoena	1727	1727_18	-87.00008	36.23154	0	0	0
AMO	pilosa	1728	1728_02	-88.04331	35.94396	99156.7	0.56851	0.6234453
AMO	deamii	1719	1719_16	-87.52286	37.18501	115939.77	0.56193	1.7926634
AMO	deamii	1721	1721_09	-88.12802	36.9404	128027.97	0.59141	1.3118596
AMO	pilosa	1714	1714_09	-86.87098	31.78844	494746.81	0.5798	1.0389322
AMO	amoena	1729	1729_59	-88.0375	35.92486	99382.99	0.33658	0.6676597
AMO	amoena	1701	1701_10	-83.14457	34.68221	389785.01	0.36717	0.6131125
AMO	deamii	1720	1720_10	-87.48447	37.14971	110978.38	0.54881	2.7703831
AMO	pilosa	1728	1728_13	-88.04331	35.94396	99156.7	0.56851	0.6234453
AMO	amoena	1715	1715_12	-85.65458	33.19064	360196.47	0.3062	1.2329308
AMO	deamii	1719	1719_20	-87.52286	37.18501	115939.77	0.56193	1.7926634
AMO	amoena	1727	1727_09	-87.00008	36.23154	0	0	0
AMO	amoena	1724	1724_10	-86.80475	36.85496	71563.35	0.17577	3.1715039
AMO	amoena	1701	1701_04	-83.14457	34.68221	389785.01	0.36717	0.6131125
AMO	deamii	1719	1719_04	-87.52286	37.18501	115939.77	0.56193	1.7926634
AMO	deamii	1719	1719_12	-87.52286	37.18501	115939.77	0.56193	1.7926634
AMO	pilosa	1718	1718_15	-88.13868	34.62799	206226.31	0.58901	1.1653841
AMO	pilosa	1714	1714_10	-86.87098	31.78844	494746.81	0.5798	1.0389322
AMO	pilosa	1713	1713_17	-89.37914	32.21889	497422.69	0.57193	1.3723948
AMO	pilosa	1713	1713_11	-89.37914	32.21889	497422.69	0.57193	1.3723948
AMO	deamii	1720	1720_10	-87.48447	37.14971	110978.38	0.54881	2.7703831
AMO	pilosa	1718	1718_11	-88.13868	34.62799	206226.31	0.58901	1.1653841
AMO	pilosa	1723	1723_03	-87.93854	36.59687	93389.65	0.5682	2.02107
AMO	pilosa	1723	1723_21	-87.93854	36.59687	93389.65	0.5682	2.02107
AMO	deamii	1720	1720_06	-87.48447	37.14971	110978.38	0.54881	2.7703831
AMO	deamii	1720	1720_05	-87.48447	37.14971	110978.38	0.54881	2.7703831
AMO	amoena	1727	1727_20	-87.00008	36.23154	0	0	0
AMO	amoena	1726	1726_11	-88.03654	35.91927	99521.44	0.33102	0.6676597
AMO	pilosa	1704	1704_01	-81.97334	30.77629	765556.29	0.65699	2.7875778

Table S3   Population distances and location (by individual)

garden	taxon	pop	genotype	longitude	latitude	geo_dist	gen_dist	env_dist
AMO	deamii	1721	1721_22	-88.12802	36.9404	128027.97	0.59141	1.3118596
AMO	pilosa	1714	1714_18	-86.87098	31.78844	494746.81	0.5798	1.0389322
AMO	pilosa	1714	1714_07	-86.87098	31.78844	494746.81	0.5798	1.0389322
AMO	pilosa	1709	1709_12	-83.42582	30.78985	690513.38	0.65094	1.872317
AMO	amoena	1715	1715_17	-85.65458	33.19064	360196.47	0.3062	1.2329308
AMO	pilosa	1714	1714_03	-86.87098	31.78844	494746.81	0.5798	1.0389322
AMO	amoena	1701	1701_16	-83.14457	34.68221	389785.01	0.36717	0.6131125
AMO	pilosa	1704	1704_18	-81.97334	30.77629	765556.29	0.65699	2.7875778
AMO	pilosa	1729	1729_01	-88.0375	35.92486	99382.99	0.54341	0.6676597
AMO	amoena	1727	1727_13	-87.00008	36.23154	0	0	0
AMO	amoena	1727	1727_16	-87.00008	36.23154	0	0	0
AMO	amoena	1716	1716_14	-87.35549	33.66987	287001.64	0.43994	1.0386371
AMO	amoena	1724	1724_17	-86.80475	36.85496	71563.35	0.17577	3.1715039
AMO	amoena	1733	1733_15	-84.39869	36.70741	238817.35	0.28783	1.1824762
AMO	deamii	1720	1720_20	-87.48447	37.14971	110978.38	0.54881	2.7703831
AMO	amoena	1733	1733_10	-84.39869	36.70741	238817.35	0.28783	1.1824762
AMO	amoena	1729	1729_65	-88.0375	35.92486	99382.99	0.33658	0.6676597
AMO	amoena	1726	1726_01	-88.03654	35.91927	99521.44	0.33102	0.6676597
AMO	deamii	1721	1721_21	-88.12802	36.9404	128027.97	0.59141	1.3118596
AMO	deamii	1721	1721_22	-88.12802	36.9404	128027.97	0.59141	1.3118596
AMO	pilosa	1718	1718_06	-88.13868	34.62799	206226.31	0.58901	1.1653841
AMO	amoena	1716	1716_04	-87.35549	33.66987	287001.64	0.43994	1.0386371
AMO	deamii	1603	1603_BG56	-87.4962	37.09251	105585.74	0.54881	2.7703831
AMO	pilosa	1713	1713_05	-89.37914	32.21889	497422.69	0.57193	1.3723948
AMO	pilosa	1713	1713_01	-89.37914	32.21889	497422.69	0.57193	1.3723948
AMO	amoena	1716	1716_16	-87.35549	33.66987	287001.64	0.43994	1.0386371
AMO	deamii	1505	1505_BG16	-87.4839	37.15035	111024.47	0.54881	2.7703831
AMO	amoena	1726	1726_07	-88.03654	35.91927	99521.44	0.33102	0.6676597
AMO	pilosa	1729	1729_02	-88.0375	35.92486	99382.99	0.54341	0.6676597
AMO	pilosa	1723	1723_18	-87.93854	36.59687	93389.65	0.5682	2.02107
AMO	pilosa	1729	1729_11	-88.0375	35.92486	99382.99	0.54341	0.6676597
AMO	amoena	1729	1729_53	-88.0375	35.92486	99382.99	0.33658	0.6676597
AMO	amoena	1727	1727_03	-87.00008	36.23154	0	0	0
AMO	amoena	1715	1715_07	-85.65458	33.19064	360196.47	0.3062	1.2329308
AMO	deamii	1720	1720_08	-87.48447	37.14971	110978.38	0.54881	2.7703831
AMO	amoena	1729	1729_62	-88.0375	35.92486	99382.99	0.33658	0.6676597
AMO	deamii	1719	1719_04	-87.52286	37.18501	115939.77	0.56193	1.7926634
AMO	amoena	1716	1716_10	-87.35549	33.66987	287001.64	0.43994	1.0386371
AMO	pilosa	1712	1712_12	-89.90611	30.91547	650175.32	0.59966	1.5227537
AMO	amoena	1724	1724_03	-86.80475	36.85496	71563.35	0.17577	3.1715039
AMO	pilosa	1723	1723_13	-87.93854	36.59687	93389.65	0.5682	2.02107
AMO	deamii	1719	1719_13	-87.52286	37.18501	115939.77	0.56193	1.7926634
AMO	pilosa	1729	1729_16	-88.0375	35.92486	99382.99	0.54341	0.6676597
AMO	amoena	1715	1715_05	-85.65458	33.19064	360196.47	0.3062	1.2329308
AMO	pilosa	1718	1718_01	-88.13868	34.62799	206226.31	0.58901	1.1653841
AMO	pilosa	1723	1723_09	-87.93854	36.59687	93389.65	0.5682	2.02107
AMO	pilosa	1712	1712_05	-89.90611	30.91547	650175.32	0.59966	1.5227537
AMO	deamii	1720	1720_17	-87.48447	37.14971	110978.38	0.54881	2.7703831
AMO	amoena	1726	1726_21	-88.03654	35.91927	99521.44	0.33102	0.6676597
AMO	amoena	1724	1724_09	-86.80475	36.85496	71563.35	0.17577	3.1715039
AMO	deamii	1721	1721_09	-88.12802	36.9404	128027.97	0.59141	1.3118596
AMO	amoena	1729	1729_58	-88.0375	35.92486	99382.99	0.33658	0.6676597

Table S3 Population distances and location (by individual)

garden	taxon	pop	genotype	longitude	latitude	geo_dist	gen_dist	env_dist
AMO	pilosa	1709	1709_08	-83.42582	30.78985	690513.38	0.65094	1.872317
AMO	deamii	1720	1720_01	-87.48447	37.14971	110978.38	0.54881	2.7703831
AMO	pilosa	1728	1728_06	-88.04331	35.94396	99156.7	0.56851	0.6234453
AMO	pilosa	1728	1728_04	-88.04331	35.94396	99156.7	0.56851	0.6234453
AMO	pilosa	1728	1728_01	-88.04331	35.94396	99156.7	0.56851	0.6234453
AMO	pilosa	1718	1718_16	-88.13868	34.62799	206226.31	0.58901	1.1653841
AMO	amoena	1701	1701_13	-83.14457	34.68221	389785.01	0.36717	0.6131125
AMO	pilosa	1713	1713_08	-89.37914	32.21889	497422.69	0.57193	1.3723948
AMO	amoena	1727	1727_11	-87.00008	36.23154	0	0	0
AMO	amoena	1729	1729_63	-88.0375	35.92486	99382.99	0.33658	0.6676597
AMO	amoena	1716	1716_19	-87.35549	33.66987	287001.64	0.43994	1.0386371
AMO	amoena	1724	1724_19	-86.80475	36.85496	71563.35	0.17577	3.1715039
AMO	amoena	1701	1701_11	-83.14457	34.68221	389785.01	0.36717	0.6131125
AMO	amoena	1726	1726_08	-88.03654	35.91927	99521.44	0.33102	0.6676597
AMO	amoena	1716	1716_02	-87.35549	33.66987	287001.64	0.43994	1.0386371
AMO	deamii	1719	1719_18	-87.52286	37.18501	115939.77	0.56193	1.7926634
AMO	pilosa	1728	1728_03	-88.04331	35.94396	99156.7	0.56851	0.6234453
AMO	amoena	1724	1724_07	-86.80475	36.85496	71563.35	0.17577	3.1715039
AMO	deamii	1719	1719_06	-87.52286	37.18501	115939.77	0.56193	1.7926634
AMO	amoena	1701	1701_20	-83.14457	34.68221	389785.01	0.36717	0.6131125
AMO	deamii	1721	1721_21	-88.12802	36.9404	128027.97	0.59141	1.3118596
AMO	pilosa	1718	1718_02	-88.13868	34.62799	206226.31	0.58901	1.1653841
AMO	amoena	1724	1724_05	-86.80475	36.85496	71563.35	0.17577	3.1715039
AMO	pilosa	1729	1729_04	-88.0375	35.92486	99382.99	0.54341	0.6676597
AMO	amoena	1726	1726_14	-88.03654	35.91927	99521.44	0.33102	0.6676597
AMO	deamii	1719	1719_06	-87.52286	37.18501	115939.77	0.56193	1.7926634
AMO	amoena	1715	1715_16	-85.65458	33.19064	360196.47	0.3062	1.2329308
AMO	pilosa	1712	1712_15	-89.90611	30.91547	650175.32	0.59966	1.5227537
AMO	pilosa	1713	1713_02	-89.37914	32.21889	497422.69	0.57193	1.3723948
AMO	deamii	1719	1719_11	-87.52286	37.18501	115939.77	0.56193	1.7926634
AMO	pilosa	1708	1708_08	-83.52907	30.8033	684636.29	0.65094	1.872317
AMO	amoena	1733	1733_16	-84.39869	36.70741	238817.35	0.28783	1.1824762
AMO	pilosa	1728	1728_11	-88.04331	35.94396	99156.7	0.56851	0.6234453
AMO	deamii	1720	1720_08	-87.48447	37.14971	110978.38	0.54881	2.7703831
AMO	pilosa	1728	1728_24	-88.04331	35.94396	99156.7	0.56851	0.6234453
AMO	deamii	1721	1721_13	-88.12802	36.9404	128027.97	0.59141	1.3118596
AMO	amoena	1715	1715_04	-85.65458	33.19064	360196.47	0.3062	1.2329308
AMO	deamii	1505	1505_BG17	-87.4839	37.15035	111024.47	0.54881	2.7703831
AMO	amoena	1729	1729_56	-88.0375	35.92486	99382.99	0.33658	0.6676597
AMO	amoena	1729	1729_49	-88.0375	35.92486	99382.99	0.33658	0.6676597
AMO	pilosa	1729	1729_15	-88.0375	35.92486	99382.99	0.54341	0.6676597
AMO	deamii	1720	1720_05	-87.48447	37.14971	110978.38	0.54881	2.7703831
AMO	pilosa	1712	1712_01	-89.90611	30.91547	650175.32	0.59966	1.5227537
AMO	amoena	1727	1727_08	-87.00008	36.23154	0	0	0
AMO	pilosa	1728	1728_19	-88.04331	35.94396	99156.7	0.56851	0.6234453
AMO	pilosa	1713	1713_04	-89.37914	32.21889	497422.69	0.57193	1.3723948
AMO	pilosa	1714	1714_17	-86.87098	31.78844	494746.81	0.5798	1.0389322
AMO	pilosa	1723	1723_04	-87.93854	36.59687	93389.65	0.5682	2.02107
AMO	amoena	1701	1701_07	-83.14457	34.68221	389785.01	0.36717	0.6131125
AMO	deamii	1504	1504_BG15	-87.4961	37.0928	105611.01	0.54881	2.7703831
AMO	deamii	1720	1720_07	-87.48447	37.14971	110978.38	0.54881	2.7703831
AMO	amoena	1715	1715_13	-85.65458	33.19064	360196.47	0.3062	1.2329308

Table S3 Population distances and location (by individual)

garden	taxon	pop	genotype	longitude	latitude	geo_dist	gen_dist	env_dist
AMO	pilosa	1718	1718_07	-88.13868	34.62799	206226.31	0.58901	1.1653841
AMO	pilosa	1714	1714_01	-86.87098	31.78844	494746.81	0.5798	1.0389322
AMO	amoena	1727	1727_17	-87.00008	36.23154	0	0	0
AMO	deamii	1719	1719_18	-87.52286	37.18501	115939.77	0.56193	1.7926634
AMO	amoena	1726	1726_10	-88.03654	35.91927	99521.44	0.33102	0.6676597
AMO	pilosa	1723	1723_08	-87.93854	36.59687	93389.65	0.5682	2.02107
AMO	deamii	1721	1721_15	-88.12802	36.9404	128027.97	0.59141	1.3118596
AMO	amoena	1733	1733_02	-84.39869	36.70741	238817.35	0.28783	1.1824762
AMO	amoena	1727	1727_23	-87.00008	36.23154	0	0	0
AMO	amoena	1726	1726_04	-88.03654	35.91927	99521.44	0.33102	0.6676597
AMO	amoena	1716	1716_06	-87.35549	33.66987	287001.64	0.43994	1.0386371
AMO	deamii	1720	1720_19	-87.48447	37.14971	110978.38	0.54881	2.7703831
AMO	pilosa	1718	1718_10	-88.13868	34.62799	206226.31	0.58901	1.1653841
AMO	pilosa	1723	1723_15	-87.93854	36.59687	93389.65	0.5682	2.02107
AMO	amoena	1701	1701_02	-83.14457	34.68221	389785.01	0.36717	0.6131125
AMO	pilosa	1729	1729_20	-88.0375	35.92486	99382.99	0.54341	0.6676597
AMO	amoena	1715	1715_08	-85.65458	33.19064	360196.47	0.3062	1.2329308
AMO	pilosa	1723	1723_20	-87.93854	36.59687	93389.65	0.5682	2.02107
AMO	pilosa	1709	1709_05	-83.42582	30.78985	690513.38	0.65094	1.872317
AMO	pilosa	1723	1723_11	-87.93854	36.59687	93389.65	0.5682	2.02107
AMO	pilosa	1718	1718_13	-88.13868	34.62799	206226.31	0.58901	1.1653841
AMO	pilosa	1712	1712_11	-89.90611	30.91547	650175.32	0.59966	1.5227537
AMO	pilosa	1714	1714_08	-86.87098	31.78844	494746.81	0.5798	1.0389322
AMO	pilosa	1713	1713_15	-89.37914	32.21889	497422.69	0.57193	1.3723948
AMO	amoena	1724	1724_11	-86.80475	36.85496	71563.35	0.17577	3.1715039
AMO	deamii	1720	1720_01	-87.48447	37.14971	110978.38	0.54881	2.7703831
AMO	pilosa	1704	1704_11	-81.97334	30.77629	765556.29	0.65699	2.7875778
AMO	pilosa	1714	1714_13	-86.87098	31.78844	494746.81	0.5798	1.0389322
AMO	deamii	1719	1719_12	-87.52286	37.18501	115939.77	0.56193	1.7926634
AMO	amoena	1716	1716_13	-87.35549	33.66987	287001.64	0.43994	1.0386371
AMO	deamii	1720	1720_17	-87.48447	37.14971	110978.38	0.54881	2.7703831
AMO	amoena	1733	1733_09	-84.39869	36.70741	238817.35	0.28783	1.1824762
AMO	deamii	1721	1721_12	-88.12802	36.9404	128027.97	0.59141	1.3118596
AMO	amoena	1724	1724_13	-86.80475	36.85496	71563.35	0.17577	3.1715039
AMO	pilosa	1713	1713_06	-89.37914	32.21889	497422.69	0.57193	1.3723948
AMO	amoena	1701	1701_06	-83.14457	34.68221	389785.01	0.36717	0.6131125
AMO	deamii	1719	1719_08	-87.52286	37.18501	115939.77	0.56193	1.7926634
AMO	pilosa	1723	1723_01	-87.93854	36.59687	93389.65	0.5682	2.02107
AMO	deamii	1508	1508_BG18	-88.1283	36.9402	128033.99	0.59141	1.3118596
AMO	deamii	1603	1603_BG56	-87.4962	37.09251	105585.74	0.54881	2.7703831
AMO	deamii	1721	1721_12	-88.12802	36.9404	128027.97	0.59141	1.3118596
AMO	amoena	1701	1701_08	-83.14457	34.68221	389785.01	0.36717	0.6131125
AMO	deamii	1721	1721_19	-88.12802	36.9404	128027.97	0.59141	1.3118596
AMO	pilosa	1718	1718_17	-88.13868	34.62799	206226.31	0.58901	1.1653841
AMO	amoena	1724	1724_06	-86.80475	36.85496	71563.35	0.17577	3.1715039
AMO	pilosa	1704	1704_09	-81.97334	30.77629	765556.29	0.65699	2.7875778
AMO	pilosa	1704	1704_12	-81.97334	30.77629	765556.29	0.65699	2.7875778
AMO	deamii	1720	1720_16	-87.48447	37.14971	110978.38	0.54881	2.7703831
AMO	pilosa	1712	1712_14	-89.90611	30.91547	650175.32	0.59966	1.5227537
AMO	pilosa	1713	1713_09	-89.37914	32.21889	497422.69	0.57193	1.3723948
AMO	amoena	1716	1716_01	-87.35549	33.66987	287001.64	0.43994	1.0386371
AMO	pilosa	1728	1728_07	-88.04331	35.94396	99156.7	0.56851	0.6234453

Table S3 Population distances and location (by individual)

garden	taxon	pop	genotype	longitude	latitude	geo_dist	gen_dist	env_dist
AMO	deamii	1719	1719_01	-87.52286	37.18501	115939.77	0.56193	1.7926634
AMO	amoena	1715	1715_10	-85.65458	33.19064	360196.47	0.3062	1.2329308
AMO	amoena	1726	1726_12	-88.03654	35.91927	99521.44	0.33102	0.6676597
AMO	pilosa	1718	1718_04	-88.13868	34.62799	206226.31	0.58901	1.1653841
AMO	pilosa	1713	1713_13	-89.37914	32.21889	497422.69	0.57193	1.3723948
AMO	amoena	1729	1729_66	-88.0375	35.92486	99382.99	0.33658	0.6676597
AMO	pilosa	1709	1709_04	-83.42582	30.78985	690513.38	0.65094	1.872317
AMO	amoena	1727	1727_21	-87.00008	36.23154	0	0	0
AMO	deamii	1719	1719_19	-87.52286	37.18501	115939.77	0.56193	1.7926634
AMO	deamii	1720	1720_19	-87.48447	37.14971	110978.38	0.54881	2.7703831
AMO	pilosa	1714	1714_05	-86.87098	31.78844	494746.81	0.5798	1.0389322
AMO	amoena	1724	1724_16	-86.80475	36.85496	71563.35	0.17577	3.1715039
AMO	amoena	1715	1715_14	-85.65458	33.19064	360196.47	0.3062	1.2329308
AMO	deamii	1719	1719_13	-87.52286	37.18501	115939.77	0.56193	1.7926634
AMO	pilosa	1723	1723_22	-87.93854	36.59687	93389.65	0.5682	2.02107
AMO	pilosa	1718	1718_12	-88.13868	34.62799	206226.31	0.58901	1.1653841
AMO	amoena	1729	1729_54	-88.0375	35.92486	99382.99	0.33658	0.6676597
AMO	amoena	1729	1729_45	-88.0375	35.92486	99382.99	0.33658	0.6676597
AMO	deamii	1505	1505_BG16	-87.4839	37.15035	111024.47	0.54881	2.7703831
AMO	amoena	1726	1726_15	-88.03654	35.91927	99521.44	0.33102	0.6676597
AMO	pilosa	1723	1723_16	-87.93854	36.59687	93389.65	0.5682	2.02107
AMO	amoena	1724	1724_02	-86.80475	36.85496	71563.35	0.17577	3.1715039
AMO	deamii	1720	1720_14	-87.48447	37.14971	110978.38	0.54881	2.7703831
AMO	amoena	1701	1701_17	-83.14457	34.68221	389785.01	0.36717	0.6131125
AMO	pilosa	1718	1718_03	-88.13868	34.62799	206226.31	0.58901	1.1653841
AMO	pilosa	1729	1729_05	-88.0375	35.92486	99382.99	0.54341	0.6676597
AMO	pilosa	1712	1712_04	-89.90611	30.91547	650175.32	0.59966	1.5227537
AMO	amoena	1733	1733_20	-84.39869	36.70741	238817.35	0.28783	1.1824762
AMO	pilosa	1728	1728_23	-88.04331	35.94396	99156.7	0.56851	0.6234453
AMO	pilosa	1728	1728_05	-88.04331	35.94396	99156.7	0.56851	0.6234453
AMO	deamii	1720	1720_09	-87.48447	37.14971	110978.382	0.54881	2.7703831
AMO	pilosa	1723	1723_10	-87.93854	36.59687	93389.652	0.5682	2.02107
AMO	amoena	1716	1716_17	-87.35549	33.66987	287001.641	0.43994	1.0386371
AMO	pilosa	1729	1729_19	-88.0375	35.92486	99382.985	0.54341	0.6676597
AMO	pilosa	1712	1712_02	-89.90611	30.91547	650175.325	0.59966	1.5227537
AMO	deamii	1720	1720_02	-87.48447	37.14971	110978.382	0.54881	2.7703831
AMO	pilosa	1723	1723_05	-87.93854	36.59687	93389.652	0.5682	2.02107
AMO	deamii	1720	1720_02	-87.48447	37.14971	110978.382	0.54881	2.7703831
AMO	amoena	1727	1727_10	-87.00008	36.23154	0	0	0
AMO	pilosa	1714	1714_15	-86.87098	31.78844	494746.806	0.5798	1.0389322
AMO	deamii	1721	1721_13	-88.12802	36.9404	128027.972	0.59141	1.3118596
AMO	deamii	1508	1508_BG18	-88.1283	36.9402	128033.986	0.59141	1.3118596
AMO	deamii	1720	1720_07	-87.48447	37.14971	110978.382	0.54881	2.7703831
AMO	amoena	1727	1727_15	-87.00008	36.23154	0	0	0
AMO	amoena	1727	1727_01	-87.00008	36.23154	0	0	0
AMO	pilosa	1729	1729_10	-88.0375	35.92486	99382.985	0.54341	0.6676597
AMO	amoena	1733	1733_04	-84.39869	36.70741	238817.348	0.28783	1.1824762
AMO	amoena	1726	1726_03	-88.03654	35.91927	99521.443	0.33102	0.6676597
AMO	pilosa	1728	1728_12	-88.04331	35.94396	99156.701	0.56851	0.6234453
AMO	amoena	1715	1715_03	-85.65458	33.19064	360196.471	0.3062	1.2329308
AMO	deamii	1719	1719_16	-87.52286	37.18501	115939.766	0.56193	1.7926634
AMO	pilosa	1713	1713_19	-89.37914	32.21889	497422.691	0.57193	1.3723948

Table S3 Population distances and location (by individual)

garden	taxon	pop	genotype	longitude	latitude	geo_dist	gen_dist	env_dist
AMO	pilosa	1718	1718_08	-88.13868	34.62799	206226.307	0.58901	1.1653841
AMO	amoena	1726	1726_05	-88.03654	35.91927	99521.443	0.33102	0.6676597
AMO	pilosa	1712	1712_16	-89.90611	30.91547	650175.325	0.59966	1.5227537
AMO	amoena	1716	1716_07	-87.35549	33.66987	287001.641	0.43994	1.0386371
AMO	amoena	1715	1715_09	-85.65458	33.19064	360196.471	0.3062	1.2329308
AMO	amoena	1729	1729_61	-88.0375	35.92486	99382.985	0.33658	0.6676597
AMO	amoena	1716	1716_15	-87.35549	33.66987	287001.641	0.43994	1.0386371
AMO	amoena	1727	1727_04	-87.00008	36.23154	0	0	0
AMO	pilosa	1714	1714_11	-86.87098	31.78844	494746.806	0.5798	1.0389322
DEA	pilosa	1713	1713_02	-89.37914	32.21889	575603.791	0.58446	4.1425168
DEA	amoena	1716	1716_19	-87.35549	33.66987	387550.473	0.58789	3.476917
DEA	pilosa	1709	1709_12	-83.42582	30.78985	800793.179	0.64798	4.6270978
DEA	pilosa	1723	1723_02	-87.93854	36.59687	73636.396	0.575	0.8250415
DEA	amoena	1715	1715_02	-85.65458	33.19064	471099.665	0.54422	2.4841575
DEA	amoena	1701	1701_02	-83.14457	34.68221	477965.291	0.58122	2.7359019
DEA	amoena	1701	1701_13	-83.14457	34.68221	477965.291	0.58122	2.7359019
DEA	amoena	1716	1716_11	-87.35549	33.66987	387550.473	0.58789	3.476917
DEA	deamii	1721	1721_22	-88.12802	36.9404	61745.493	0.22095	3.3946057
DEA	pilosa	1712	1712_11	-89.90611	30.91547	728982.306	0.60627	4.0056394
DEA	pilosa	1728	1728_12	-88.04331	35.94396	143224.733	0.57849	2.9793937
DEA	deamii	1721	1721_12	-88.12802	36.9404	61745.493	0.22095	3.3946057
DEA	pilosa	1714	1714_07	-86.87098	31.78844	599458.225	0.58533	3.0630482
DEA	amoena	1724	1724_09	-86.80475	36.85496	68760.704	0.53767	0.5421331
DEA	pilosa	1718	1718_03	-88.13868	34.62799	286847.569	0.60118	3.9057865
DEA	amoena	1724	1724_19	-86.80475	36.85496	68760.704	0.53767	0.5421331
DEA	deamii	1719	1719_20	-87.52286	37.18501	5200.245	0.094	1.083696
DEA	amoena	1729	1729_62	-88.0375	35.92486	145042.89	0.58467	2.988086
DEA	deamii	1720	1720_06	-87.48447	37.14971	0	0	0
DEA	amoena	1701	1701_11	-83.14457	34.68221	477965.291	0.58122	2.7359019
DEA	amoena	1716	1716_09	-87.35549	33.66987	387550.473	0.58789	3.476917
DEA	amoena	1724	1724_10	-86.80475	36.85496	68760.704	0.53767	0.5421331
DEA	pilosa	1723	1723_13	-87.93854	36.59687	73636.396	0.575	0.8250415
DEA	amoena	1729	1729_54	-88.0375	35.92486	145042.89	0.58467	2.988086
DEA	amoena	1715	1715_11	-85.65458	33.19064	471099.665	0.54422	2.4841575
DEA	pilosa	1704	1704_12	-81.97334	30.77629	872689.662	0.65999	5.5339045
DEA	amoena	1733	1733_20	-84.39869	36.70741	278959.986	0.54028	2.3222791
DEA	deamii	1719	1719_06	-87.52286	37.18501	5200.245	0.094	1.083696
DEA	amoena	1716	1716_04	-87.35549	33.66987	387550.473	0.58789	3.476917
DEA	pilosa	1728	1728_02	-88.04331	35.94396	143224.733	0.57849	2.9793937
DEA	deamii	1719	1719_08	-87.52286	37.18501	5200.245	0.094	1.083696
DEA	pilosa	1723	1723_19	-87.93854	36.59687	73636.396	0.575	0.8250415
DEA	amoena	1729	1729_48	-88.0375	35.92486	145042.89	0.58467	2.988086
DEA	pilosa	1713	1713_04	-89.37914	32.21889	575603.791	0.58446	4.1425168
DEA	pilosa	1728	1728_11	-88.04331	35.94396	143224.733	0.57849	2.9793937
DEA	deamii	1720	1720_17	-87.48447	37.14971	0	0	0
DEA	pilosa	1718	1718_08	-88.13868	34.62799	286847.569	0.60118	3.9057865
DEA	pilosa	1729	1729_02	-88.0375	35.92486	145042.89	0.56357	2.988086
DEA	deamii	1720	1720_19	-87.48447	37.14971	0	0	0
DEA	pilosa	1714	1714_14	-86.87098	31.78844	599458.225	0.58533	3.0630482
DEA	amoena	1729	1729_66	-88.0375	35.92486	145042.89	0.58467	2.988086
DEA	deamii	1720	1720_07	-87.48447	37.14971	0	0	0
DEA	pilosa	1729	1729_01	-88.0375	35.92486	145042.89	0.56357	2.988086

Table S3 Population distances and location (by individual)

garden	taxon	pop	genotype	longitude	latitude	geo_dist	gen_dist	env_dist
DEA	pilosa	1704	1704_11	-81.97334	30.77629	872689.662	0.65999	5.5339045
DEA	deamii	1720	1720_03	-87.48447	37.14971	0	0	0
DEA	deamii	1719	1719_20	-87.52286	37.18501	5200.245	0.094	1.083696
DEA	pilosa	1723	1723_05	-87.93854	36.59687	73636.396	0.575	0.8250415
DEA	pilosa	1718	1718_05	-88.13868	34.62799	286847.569	0.60118	3.9057865
DEA	amoena	1727	1727_10	-87.00008	36.23154	110978.382	0.54881	2.7703831
DEA	amoena	1726	1726_04	-88.03654	35.91927	145600.5	0.57847	2.988086
DEA	pilosa	1723	1723_17	-87.93854	36.59687	73636.396	0.575	0.8250415
DEA	pilosa	1712	1712_15	-89.90611	30.91547	728982.306	0.60627	4.0056394
DEA	deamii	1720	1720_03	-87.48447	37.14971	0	0	0
DEA	amoena	1726	1726_08	-88.03654	35.91927	145600.5	0.57847	2.988086
DEA	pilosa	1718	1718_15	-88.13868	34.62799	286847.569	0.60118	3.9057865
DEA	deamii	1721	1721_15	-88.12802	36.9404	61745.493	0.22095	3.3946057
DEA	pilosa	1729	1729_20	-88.0375	35.92486	145042.89	0.56357	2.988086
DEA	pilosa	1729	1729_15	-88.0375	35.92486	145042.89	0.56357	2.988086
DEA	amoena	1727	1727_16	-87.00008	36.23154	110978.382	0.54881	2.7703831
DEA	amoena	1726	1726_10	-88.03654	35.91927	145600.5	0.57847	2.988086
DEA	amoena	1715	1715_14	-85.65458	33.19064	471099.665	0.54422	2.4841575
DEA	pilosa	1713	1713_14	-89.37914	32.21889	575603.791	0.58446	4.1425168
DEA	deamii	1720	1720_17	-87.48447	37.14971	0	0	0
DEA	amoena	1727	1727_21	-87.00008	36.23154	110978.382	0.54881	2.7703831
DEA	amoena	1724	1724_02	-86.80475	36.85496	68760.704	0.53767	0.5421331
DEA	pilosa	1709	1709_08	-83.42582	30.78985	800793.179	0.64798	4.6270978
DEA	pilosa	1714	1714_11	-86.87098	31.78844	599458.2254	0.58533	3.0630482
DEA	amoena	1727	1727_02	-87.00008	36.23154	110978.3824	0.54881	2.7703831
DEA	deamii	1719	1719_01	-87.52286	37.18501	5200.24455	0.094	1.083696
DEA	deamii	1721	1721_21	-88.12802	36.9404	61745.49316	0.22095	3.3946057
DEA	pilosa	1713	1713_19	-89.37914	32.21889	575603.791	0.58446	4.1425168
DEA	amoena	1733	1733_03	-84.39869	36.70741	278959.9861	0.54028	2.3222791
DEA	pilosa	1718	1718_20	-88.13868	34.62799	286847.569	0.60118	3.9057865
DEA	amoena	1701	1701_07	-83.14457	34.68221	477965.2911	0.58122	2.7359019
DEA	deamii	1508	1508_BG18	-88.1283	36.9402	61776.42325	0.22095	3.3946057
DEA	amoena	1726	1726_19	-88.03654	35.91927	145600.4998	0.57847	2.988086
DEA	amoena	1715	1715_09	-85.65458	33.19064	471099.6649	0.54422	2.4841575
DEA	amoena	1727	1727_07	-87.00008	36.23154	110978.3824	0.54881	2.7703831
DEA	pilosa	1713	1713_10	-89.37914	32.21889	575603.791	0.58446	4.1425168
DEA	deamii	1719	1719_11	-87.52286	37.18501	5200.24455	0.094	1.083696
DEA	amoena	1724	1724_11	-86.80475	36.85496	68760.70372	0.53767	0.5421331
DEA	pilosa	1723	1723_12	-87.93854	36.59687	73636.39612	0.575	0.8250415
DEA	deamii	1720	1720_01	-87.48447	37.14971	0	0	0
DEA	amoena	1716	1716_18	-87.35549	33.66987	387550.473	0.58789	3.476917
DEA	pilosa	1714	1714_15	-86.87098	31.78844	599458.2254	0.58533	3.0630482
DEA	amoena	1727	1727_18	-87.00008	36.23154	110978.3824	0.54881	2.7703831
DEA	amoena	1716	1716_10	-87.35549	33.66987	387550.473	0.58789	3.476917
DEA	deamii	1720	1720_10	-87.48447	37.14971	0	0	0
DEA	deamii	1721	1721_19	-88.12802	36.9404	61745.49316	0.22095	3.3946057
DEA	deamii	1721	1721_19	-88.12802	36.9404	61745.49316	0.22095	3.3946057
DEA	pilosa	1709	1709_06	-83.42582	30.78985	800793.1789	0.64798	4.6270978
DEA	amoena	1729	1729_45	-88.0375	35.92486	145042.8897	0.58467	2.988086
DEA	amoena	1733	1733_04	-84.39869	36.70741	278959.9861	0.54028	2.3222791
DEA	amoena	1729	1729_64	-88.0375	35.92486	145042.8897	0.58467	2.988086
DEA	amoena	1727	1727_03	-87.00008	36.23154	110978.3824	0.54881	2.7703831

Table S3 Population distances and location (by individual)

garden	taxon	pop	genotype	longitude	latitude	geo_dist	gen_dist	env_dist
DEA	deamii	1720	1720_16	-87.48447	37.14971	0	0	0
DEA	amoena	1729	1729_61	-88.0375	35.92486	145042.8897	0.58467	2.988086
DEA	pilosa	1714	1714_09	-86.87098	31.78844	599458.2254	0.58533	3.0630482
DEA	pilosa	1729	1729_19	-88.0375	35.92486	145042.8897	0.56357	2.988086
DEA	pilosa	1704	1704_10	-81.97334	30.77629	872689.6618	0.65999	5.5339045
DEA	pilosa	1713	1713_11	-89.37914	32.21889	575603.791	0.58446	4.1425168
DEA	pilosa	1713	1713_13	-89.37914	32.21889	575603.791	0.58446	4.1425168
DEA	amoena	1715	1715_07	-85.65458	33.19064	471099.6649	0.54422	2.4841575
DEA	deamii	1504	1504_BG15	-87.4961	37.0928	6418.43074	0	0
DEA	pilosa	1723	1723_18	-87.93854	36.59687	73636.39612	0.575	0.8250415
DEA	pilosa	1729	1729_22	-88.0375	35.92486	145042.8897	0.56357	2.988086
DEA	pilosa	1728	1728_03	-88.04331	35.94396	143224.7334	0.57849	2.9793937
DEA	amoena	1716	1716_03	-87.35549	33.66987	387550.473	0.58789	3.476917
DEA	deamii	1505	1505_BG16	-87.4839	37.15035	87.59184	0	0
DEA	deamii	1720	1720_16	-87.48447	37.14971	0	0	0
DEA	amoena	1727	1727_04	-87.00008	36.23154	110978.3824	0.54881	2.7703831
DEA	pilosa	1718	1718_11	-88.13868	34.62799	286847.569	0.60118	3.9057865
DEA	deamii	1720	1720_05	-87.48447	37.14971	0	0	0
DEA	amoena	1724	1724_03	-86.80475	36.85496	68760.70372	0.53767	0.5421331
DEA	pilosa	1728	1728_25	-88.04331	35.94396	143224.7334	0.57849	2.9793937
DEA	deamii	1719	1719_19	-87.52286	37.18501	5200.24455	0.094	1.083696
DEA	amoena	1726	1726_12	-88.03654	35.91927	145600.4998	0.57847	2.988086
DEA	pilosa	1714	1714_05	-86.87098	31.78844	599458.2254	0.58533	3.0630482
DEA	deamii	1720	1720_07	-87.48447	37.14971	0	0	0
DEA	amoena	1701	1701_08	-83.14457	34.68221	477965.2911	0.58122	2.7359019
DEA	amoena	1701	1701_06	-83.14457	34.68221	477965.2911	0.58122	2.7359019
DEA	pilosa	1723	1723_03	-87.93854	36.59687	73636.39612	0.575	0.8250415
DEA	deamii	1603	1603_BG56	-87.4962	37.09251	6451.22532	0	0
DEA	pilosa	1712	1712_14	-89.90611	30.91547	728982.3063	0.60627	4.0056394
DEA	pilosa	1729	1729_11	-88.0375	35.92486	145042.8897	0.56357	2.988086
DEA	amoena	1726	1726_03	-88.03654	35.91927	145600.4998	0.57847	2.988086
DEA	pilosa	1713	1713_06	-89.37914	32.21889	575603.791	0.58446	4.1425168
DEA	amoena	1727	1727_15	-87.00008	36.23154	110978.3824	0.54881	2.7703831
DEA	deamii	1719	1719_04	-87.52286	37.18501	5200.24455	0.094	1.083696
DEA	pilosa	1714	1714_03	-86.87098	31.78844	599458.2254	0.58533	3.0630482
DEA	amoena	1715	1715_03	-85.65458	33.19064	471099.6649	0.54422	2.4841575
DEA	deamii	1719	1719_01	-87.52286	37.18501	5200.24455	0.094	1.083696
DEA	pilosa	1718	1718_06	-88.13868	34.62799	286847.569	0.60118	3.9057865
DEA	pilosa	1718	1718_01	-88.13868	34.62799	286847.569	0.60118	3.9057865
DEA	pilosa	1723	1723_09	-87.93854	36.59687	73636.39612	0.575	0.8250415
DEA	pilosa	1729	1729_04	-88.0375	35.92486	145042.8897	0.56357	2.988086
DEA	pilosa	1728	1728_01	-88.04331	35.94396	143224.7334	0.57849	2.9793937
DEA	amoena	1726	1726_21	-88.03654	35.91927	145600.4998	0.57847	2.988086
DEA	deamii	1719	1719_13	-87.52286	37.18501	5200.24455	0.094	1.083696
DEA	pilosa	1728	1728_08	-88.04331	35.94396	143224.7334	0.57849	2.9793937
DEA	pilosa	1704	1704_18	-81.97334	30.77629	872689.6618	0.65999	5.5339045
DEA	deamii	1720	1720_20	-87.48447	37.14971	0	0	0
DEA	amoena	1716	1716_13	-87.35549	33.66987	387550.473	0.58789	3.476917
DEA	deamii	1720	1720_20	-87.48447	37.14971	0	0	0
DEA	pilosa	1718	1718_14	-88.13868	34.62799	286847.569	0.60118	3.9057865
DEA	amoena	1701	1701_17	-83.14457	34.68221	477965.2911	0.58122	2.7359019
DEA	amoena	1715	1715_17	-85.65458	33.19064	471099.6649	0.54422	2.4841575

Table S3 Population distances and location (by individual)

garden	taxon	pop	genotype	longitude	latitude	geo_dist	gen_dist	env_dist
DEA	pilosa	1728	1728_13	-88.04331	35.94396	143224.7334	0.57849	2.9793937
DEA	amoena	1733	1733_15	-84.39869	36.70741	278959.9861	0.54028	2.3222791
DEA	pilosa	1712	1712_01	-89.90611	30.91547	728982.3063	0.60627	4.0056394
DEA	pilosa	1712	1712_05	-89.90611	30.91547	728982.3063	0.60627	4.0056394
DEA	deamii	1720	1720_02	-87.48447	37.14971	0	0	0
DEA	amoena	1715	1715_12	-85.65458	33.19064	471099.6649	0.54422	2.4841575
DEA	amoena	1724	1724_08	-86.80475	36.85496	68760.70372	0.53767	0.5421331
DEA	amoena	1727	1727_13	-87.00008	36.23154	110978.3824	0.54881	2.7703831
DEA	pilosa	1723	1723_20	-87.93854	36.59687	73636.39612	0.575	0.8250415
DEA	deamii	1719	1719_13	-87.52286	37.18501	5200.24455	0.094	1.083696
DEA	amoena	1724	1724_17	-86.80475	36.85496	68760.70372	0.53767	0.5421331
DEA	amoena	1729	1729_58	-88.0375	35.92486	145042.8897	0.58467	2.988086
DEA	pilosa	1712	1712_16	-89.90611	30.91547	728982.3063	0.60627	4.0056394
DEA	amoena	1726	1726_05	-88.03654	35.91927	145600.4998	0.57847	2.988086
DEA	deamii	1720	1720_14	-87.48447	37.14971	0	0	0
DEA	pilosa	1714	1714_10	-86.87098	31.78844	599458.2254	0.58533	3.0630482
DEA	amoena	1701	1701_04	-83.14457	34.68221	477965.2911	0.58122	2.7359019
DEA	amoena	1726	1726_15	-88.03654	35.91927	145600.4998	0.57847	2.988086
DEA	amoena	1729	1729_63	-88.0375	35.92486	145042.8897	0.58467	2.988086
DEA	amoena	1733	1733_10	-84.39869	36.70741	278959.9861	0.54028	2.3222791
DEA	amoena	1701	1701_18	-83.14457	34.68221	477965.2911	0.58122	2.7359019
DEA	pilosa	1712	1712_04	-89.90611	30.91547	728982.3063	0.60627	4.0056394
DEA	pilosa	1723	1723_10	-87.93854	36.59687	73636.39612	0.575	0.8250415
DEA	amoena	1715	1715_05	-85.65458	33.19064	471099.6649	0.54422	2.4841575
DEA	deamii	1719	1719_06	-87.52286	37.18501	5200.24455	0.094	1.083696
DEA	deamii	1508	1508_BG18	-88.1283	36.9402	61776.42325	0.22095	3.3946057
DEA	amoena	1724	1724_06	-86.80475	36.85496	68760.70372	0.53767	0.5421331
DEA	deamii	1719	1719_16	-87.52286	37.18501	5200.24455	0.094	1.083696
DEA	pilosa	1712	1712_03	-89.90611	30.91547	728982.3063	0.60627	4.0056394
DEA	deamii	1720	1720_08	-87.48447	37.14971	0	0	0
DEA	deamii	1505	1505_BG17	-87.4839	37.15035	87.59184	0	0
DEA	amoena	1727	1727_17	-87.00008	36.23154	110978.3824	0.54881	2.7703831
DEA	amoena	1715	1715_13	-85.65458	33.19064	471099.6649	0.54422	2.4841575
DEA	deamii	1720	1720_19	-87.48447	37.14971	0	0	0
DEA	amoena	1701	1701_10	-83.14457	34.68221	477965.2911	0.58122	2.7359019
DEA	amoena	1727	1727_09	-87.00008	36.23154	110978.3824	0.54881	2.7703831
DEA	amoena	1715	1715_15	-85.65458	33.19064	471099.6649	0.54422	2.4841575
DEA	deamii	1720	1720_02	-87.48447	37.14971	0	0	0
DEA	pilosa	1718	1718_07	-88.13868	34.62799	286847.569	0.60118	3.9057865
DEA	deamii	1721	1721_09	-88.12802	36.9404	61745.49316	0.22095	3.3946057
DEA	amoena	1727	1727_20	-87.00008	36.23154	110978.3824	0.54881	2.7703831
DEA	pilosa	1723	1723_07	-87.93854	36.59687	73636.39612	0.575	0.8250415
DEA	pilosa	1729	1729_16	-88.0375	35.92486	145042.8897	0.56357	2.988086
DEA	pilosa	1728	1728_17	-88.04331	35.94396	143224.7334	0.57849	2.9793937
DEA	pilosa	1729	1729_10	-88.0375	35.92486	145042.8897	0.56357	2.988086
DEA	pilosa	1728	1728_07	-88.04331	35.94396	143224.7334	0.57849	2.9793937
DEA	pilosa	1723	1723_04	-87.93854	36.59687	73636.39612	0.575	0.8250415
DEA	amoena	1724	1724_16	-86.80475	36.85496	68760.70372	0.53767	0.5421331
DEA	deamii	1719	1719_16	-87.52286	37.18501	5200.24455	0.094	1.083696
DEA	amoena	1729	1729_65	-88.0375	35.92486	145042.8897	0.58467	2.988086
DEA	deamii	1720	1720_06	-87.48447	37.14971	0	0	0
DEA	amoena	1716	1716_14	-87.35549	33.66987	387550.473	0.58789	3.476917

Table S3 Population distances and location (by individual)

garden	taxon	pop	genotype	longitude	latitude	geo_dist	gen_dist	env_dist
DEA	deamii	1721	1721_09	-88.12802	36.9404	61745.49316	0.22095	3.3946057
DEA	amoena	1727	1727_01	-87.00008	36.23154	110978.3824	0.54881	2.7703831
DEA	pilosa	1713	1713_17	-89.37914	32.21889	575603.791	0.58446	4.1425168
DEA	pilosa	1718	1718_12	-88.13868	34.62799	286847.569	0.60118	3.9057865
DEA	amoena	1726	1726_13	-88.03654	35.91927	145600.4998	0.57847	2.988086
DEA	deamii	1719	1719_11	-87.52286	37.18501	5200.24455	0.094	1.083696
DEA	amoena	1727	1727_11	-87.00008	36.23154	110978.3824	0.54881	2.7703831
DEA	amoena	1716	1716_17	-87.35549	33.66987	387550.473	0.58789	3.476917
DEA	deamii	1719	1719_19	-87.52286	37.18501	5200.24455	0.094	1.083696
DEA	pilosa	1709	1709_04	-83.42582	30.78985	800793.1789	0.64798	4.6270978
DEA	pilosa	1728	1728_23	-88.04331	35.94396	143224.7334	0.57849	2.9793937
DEA	deamii	1505	1505_BG17	-87.4839	37.15035	87.59184	0	0
DEA	amoena	1726	1726_01	-88.03654	35.91927	145600.4998	0.57847	2.988086
DEA	amoena	1715	1715_08	-85.65458	33.19064	471099.6649	0.54422	2.4841575
DEA	amoena	1724	1724_15	-86.80475	36.85496	68760.70372	0.53767	0.5421331
DEA	pilosa	1729	1729_06	-88.0375	35.92486	145042.8897	0.56357	2.988086
DEA	pilosa	1723	1723_21	-87.93854	36.59687	73636.39612	0.575	0.8250415
DEA	amoena	1716	1716_07	-87.35549	33.66987	387550.473	0.58789	3.476917
DEA	pilosa	1718	1718_09	-88.13868	34.62799	286847.569	0.60118	3.9057865
DEA	amoena	1726	1726_07	-88.03654	35.91927	145600.4998	0.57847	2.988086
DEA	pilosa	1723	1723_16	-87.93854	36.59687	73636.39612	0.575	0.8250415
DEA	deamii	1721	1721_22	-88.12802	36.9404	61745.49316	0.22095	3.3946057
DEA	amoena	1729	1729_56	-88.0375	35.92486	145042.8897	0.58467	2.988086
DEA	pilosa	1713	1713_07	-89.37914	32.21889	575603.791	0.58446	4.1425168
DEA	pilosa	1708	1708_01	-83.52907	30.8033	795046.0369	0.64798	4.6270978
DEA	pilosa	1713	1713_09	-89.37914	32.21889	575603.791	0.58446	4.1425168
DEA	deamii	1719	1719_04	-87.52286	37.18501	5200.24455	0.094	1.083696
DEA	amoena	1729	1729_53	-88.0375	35.92486	145042.8897	0.58467	2.988086
DEA	pilosa	1714	1714_18	-86.87098	31.78844	599458.2254	0.58533	3.0630482
DEA	amoena	1724	1724_04	-86.80475	36.85496	68760.70372	0.53767	0.5421331
DEA	deamii	1721	1721_13	-88.12802	36.9404	61745.49316	0.22095	3.3946057
DEA	amoena	1716	1716_01	-87.35549	33.66987	387550.473	0.58789	3.476917
DEA	pilosa	1714	1714_01	-86.87098	31.78844	599458.2254	0.58533	3.0630482
DEA	pilosa	1718	1718_17	-88.13868	34.62799	286847.569	0.60118	3.9057865
DEA	deamii	1720	1720_08	-87.48447	37.14971	0	0	0
DEA	pilosa	1713	1713_01	-89.37914	32.21889	575603.791	0.58446	4.1425168
DEA	pilosa	1718	1718_02	-88.13868	34.62799	286847.569	0.60118	3.9057865
DEA	amoena	1733	1733_16	-84.39869	36.70741	278959.9861	0.54028	2.3222791
DEA	pilosa	1728	1728_04	-88.04331	35.94396	143224.7334	0.57849	2.9793937
DEA	pilosa	1714	1714_08	-86.87098	31.78844	599458.2254	0.58533	3.0630482
DEA	pilosa	1712	1712_12	-89.90611	30.91547	728982.3063	0.60627	4.0056394
DEA	pilosa	1704	1704_09	-81.97334	30.77629	872689.6618	0.65999	5.5339045
DEA	amoena	1724	1724_05	-86.80475	36.85496	68760.70372	0.53767	0.5421331
DEA	deamii	1720	1720_09	-87.48447	37.14971	0	0	0
DEA	deamii	1720	1720_01	-87.48447	37.14971	0	0	0
DEA	pilosa	1714	1714_02	-86.87098	31.78844	599458.2254	0.58533	3.0630482
DEA	pilosa	1713	1713_03	-89.37914	32.21889	575603.791	0.58446	4.1425168
DEA	pilosa	1712	1712_13	-89.90611	30.91547	728982.3063	0.60627	4.0056394
DEA	pilosa	1728	1728_24	-88.04331	35.94396	143224.7334	0.57849	2.9793937
DEA	amoena	1733	1733_02	-84.39869	36.70741	278959.9861	0.54028	2.3222791
DEA	amoena	1715	1715_04	-85.65458	33.19064	471099.6649	0.54422	2.4841575
DEA	amoena	1726	1726_09	-88.03654	35.91927	145600.4998	0.57847	2.988086

Table S3 Population distances and location (by individual)

garden	taxon	pop	genotype	longitude	latitude	geo_dist	gen_dist	env_dist
DEA	pilosa	1728	1728_06	-88.04331	35.94396	143224.7334	0.57849	2.9793937
DEA	amoena	1724	1724_07	-86.80475	36.85496	68760.70372	0.53767	0.5421331
DEA	amoena	1701	1701_01	-83.14457	34.68221	477965.2911	0.58122	2.7359019
DEA	amoena	1733	1733_11	-84.39869	36.70741	278959.9861	0.54028	2.3222791
DEA	pilosa	1714	1714_17	-86.87098	31.78844	599458.2254	0.58533	3.0630482
DEA	pilosa	1708	1708_08	-83.52907	30.8033	795046.0369	0.64798	4.6270978
DEA	deamii	1505	1505_BG16	-87.4839	37.15035	87.59184	0	0
DEA	deamii	1603	1603_BG56	-87.4962	37.09251	6451.22532	0	0
DEA	amoena	1701	1701_05	-83.14457	34.68221	477965.2911	0.58122	2.7359019
DEA	pilosa	1714	1714_13	-86.87098	31.78844	599458.2254	0.58533	3.0630482
DEA	pilosa	1704	1704_01	-81.97334	30.77629	872689.6618	0.65999	5.5339045
DEA	amoena	1716	1716_06	-87.35549	33.66987	387550.473	0.58789	3.476917
DEA	deamii	1719	1719_08	-87.52286	37.18501	5200.24455	0.094	1.083696
DEA	amoena	1701	1701_20	-83.14457	34.68221	477965.2911	0.58122	2.7359019
DEA	pilosa	1723	1723_15	-87.93854	36.59687	73636.39612	0.575	0.8250415
DEA	amoena	1727	1727_12	-87.00008	36.23154	110978.3824	0.54881	2.7703831
DEA	deamii	1721	1721_12	-88.12802	36.9404	61745.49316	0.22095	3.3946057
DEA	amoena	1729	1729_59	-88.0375	35.92486	145042.8897	0.58467	2.988086
DEA	amoena	1716	1716_02	-87.35549	33.66987	387550.473	0.58789	3.476917
DEA	amoena	1733	1733_09	-84.39869	36.70741	278959.9861	0.54028	2.3222791
DEA	deamii	1721	1721_21	-88.12802	36.9404	61745.49316	0.22095	3.3946057
DEA	pilosa	1714	1714_19	-86.87098	31.78844	599458.2254	0.58533	3.0630482
DEA	deamii	1721	1721_13	-88.12802	36.9404	61745.49316	0.22095	3.3946057
DEA	deamii	1720	1720_05	-87.48447	37.14971	0	0	0
DEA	amoena	1701	1701_16	-83.14457	34.68221	477965.2911	0.58122	2.7359019
DEA	pilosa	1709	1709_05	-83.42582	30.78985	800793.1789	0.64798	4.6270978
DEA	amoena	1727	1727_08	-87.00008	36.23154	110978.3824	0.54881	2.7703831
DEA	amoena	1715	1715_10	-85.65458	33.19064	471099.6649	0.54422	2.4841575
DEA	pilosa	1729	1729_17	-88.0375	35.92486	145042.8897	0.56357	2.988086
DEA	pilosa	1723	1723_22	-87.93854	36.59687	73636.39612	0.575	0.8250415
DEA	deamii	1721	1721_15	-88.12802	36.9404	61745.49316	0.22095	3.3946057
DEA	amoena	1724	1724_13	-86.80475	36.85496	68760.70372	0.53767	0.5421331
DEA	amoena	1701	1701_12	-83.14457	34.68221	477965.2911	0.58122	2.7359019
DEA	deamii	1504	1504_BG15	-87.4961	37.0928	6418.43074	0	0
DEA	amoena	1715	1715_16	-85.65458	33.19064	471099.6649	0.54422	2.4841575
DEA	deamii	1720	1720_10	-87.48447	37.14971	0	0	0
DEA	pilosa	1713	1713_15	-89.37914	32.21889	575603.791	0.58446	4.1425168
DEA	pilosa	1718	1718_13	-88.13868	34.62799	286847.569	0.60118	3.9057865
DEA	deamii	1720	1720_14	-87.48447	37.14971	0	0	0
DEA	pilosa	1729	1729_12	-88.0375	35.92486	145042.8897	0.56357	2.988086
DEA	amoena	1727	1727_23	-87.00008	36.23154	110978.3824	0.54881	2.7703831
DEA	deamii	1720	1720_09	-87.48447	37.14971	0	0	0
DEA	deamii	1719	1719_18	-87.52286	37.18501	5200.24455	0.094	1.083696
DEA	amoena	1729	1729_49	-88.0375	35.92486	145042.8897	0.58467	2.988086
DEA	pilosa	1714	1714_04	-86.87098	31.78844	599458.2254	0.58533	3.0630482
DEA	amoena	1729	1729_57	-88.0375	35.92486	145042.8897	0.58467	2.988086
DEA	amoena	1724	1724_18	-86.80475	36.85496	68760.70372	0.53767	0.5421331
DEA	amoena	1726	1726_14	-88.03654	35.91927	145600.4998	0.57847	2.988086
DEA	pilosa	1723	1723_08	-87.93854	36.59687	73636.39612	0.575	0.8250415
DEA	deamii	1719	1719_12	-87.52286	37.18501	5200.24455	0.094	1.083696
DEA	amoena	1726	1726_02	-88.03654	35.91927	145600.4998	0.57847	2.988086
DEA	deamii	1719	1719_12	-87.52286	37.18501	5200.24455	0.094	1.083696

Table S3 Population distances and location (by individual)

garden	taxon	pop	genotype	longitude	latitude	geo_dist	gen_dist	env_dist
DEA	pilosa	1712	1712_09	-89.90611	30.91547	728982.3063	0.60627	4.0056394
DEA	pilosa	1718	1718_10	-88.13868	34.62799	286847.569	0.60118	3.9057865
DEA	amoena	1715	1715_06	-85.65458	33.19064	471099.6649	0.54422	2.4841575
DEA	pilosa	1713	1713_08	-89.37914	32.21889	575603.791	0.58446	4.1425168
DEA	pilosa	1718	1718_04	-88.13868	34.62799	286847.569	0.60118	3.9057865
DEA	amoena	1726	1726_11	-88.03654	35.91927	145600.4998	0.57847	2.988086
DEA	amoena	1716	1716_15	-87.35549	33.66987	387550.473	0.58789	3.476917
DEA	pilosa	1713	1713_05	-89.37914	32.21889	575603.791	0.58446	4.1425168
DEA	pilosa	1728	1728_19	-88.04331	35.94396	143224.733	0.57849	2.9793937
DEA	pilosa	1729	1729_05	-88.0375	35.92486	145042.89	0.56357	2.988086
DEA	amoena	1716	1716_16	-87.35549	33.66987	387550.473	0.58789	3.476917
DEA	deamii	1719	1719_18	-87.52286	37.18501	5200.245	0.094	1.083696
DEA	amoena	1727	1727_14	-87.00008	36.23154	110978.382	0.54881	2.7703831
DEA	pilosa	1723	1723_01	-87.93854	36.59687	73636.396	0.575	0.8250415
DEA	pilosa	1728	1728_05	-88.04331	35.94396	143224.733	0.57849	2.9793937
DEA	pilosa	1712	1712_02	-89.90611	30.91547	728982.306	0.60627	4.0056394
DEA	pilosa	1723	1723_11	-87.93854	36.59687	73636.396	0.575	0.8250415
DEA	pilosa	1718	1718_16	-88.13868	34.62799	286847.569	0.60118	3.9057865
PIL	amoena	1716	1716_16	-87.35549	33.66987	377824.693	0.60086	3.4279444
PIL	amoena	1724	1724_05	-86.80475	36.85496	115853.077	0.55297	0.7101916
PIL	pilosa	1718	1718_05	-88.13868	34.62799	265236.246	0.27097	3.6230254
PIL	deamii	1719	1719_01	-87.52286	37.18501	54156.506	0.57941	0.7799909
PIL	amoena	1701	1701_06	-83.14457	34.68221	516105.483	0.59444	2.6902371
PIL	amoena	1715	1715_04	-85.65458	33.19064	479624.113	0.55779	2.6120227
PIL	deamii	1505	1505_BG17	-87.4839	37.15035	56221.306	0.575	0.7051013
PIL	pilosa	1712	1712_11	-89.90611	30.91547	698798.812	0.35418	3.9627944
PIL	amoena	1716	1716_01	-87.35549	33.66987	377824.693	0.60086	3.4279444
PIL	pilosa	1729	1729_06	-88.0375	35.92486	120934.179	0.17792	2.9325467
PIL	deamii	1721	1721_21	-88.12802	36.9404	8415.124	0.61006	2.9327668
PIL	amoena	1724	1724_19	-86.80475	36.85496	115853.077	0.55297	0.7101916
PIL	pilosa	1709	1709_05	-83.42582	30.78985	815434.794	0.45319	4.3437356
PIL	amoena	1727	1727_04	-87.00008	36.23154	130520.466	0.5682	2.5669943
PIL	pilosa	1723	1723_20	-87.93854	36.59687	48020.651	0	0.5985479
PIL	deamii	1720	1720_02	-87.48447	37.14971	56153.158	0.575	0.7051013
PIL	pilosa	1704	1704_01	-81.97334	30.77629	894671.612	0.464	5.225737
PIL	deamii	1719	1719_13	-87.52286	37.18501	54156.506	0.57941	0.7799909
PIL	pilosa	1713	1713_06	-89.37914	32.21889	546240.51	0.31175	3.904977
PIL	deamii	1719	1719_16	-87.52286	37.18501	54156.506	0.57941	0.7799909
PIL	pilosa	1714	1714_03	-86.87098	31.78844	592004.221	0.34529	2.6268733
PIL	pilosa	1728	1728_19	-88.04331	35.94396	118789.898	0.1798	2.9135217
PIL	amoena	1726	1726_15	-88.03654	35.91927	121560.312	0.59236	2.9325467
PIL	pilosa	1714	1714_17	-86.87098	31.78844	592004.221	0.34529	2.6268733
PIL	amoena	1729	1729_58	-88.0375	35.92486	120934.179	0.60118	2.9325467
PIL	amoena	1724	1724_15	-86.80475	36.85496	115853.077	0.55297	0.7101916
PIL	deamii	1721	1721_15	-88.12802	36.9404	8415.124	0.61006	2.9327668
PIL	deamii	1720	1720_10	-87.48447	37.14971	56153.158	0.575	0.7051013
PIL	amoena	1716	1716_06	-87.35549	33.66987	377824.693	0.60086	3.4279444
PIL	pilosa	1723	1723_15	-87.93854	36.59687	48020.651	0	0.5985479
PIL	deamii	1720	1720_07	-87.48447	37.14971	56153.158	0.575	0.7051013
PIL	amoena	1727	1727_13	-87.00008	36.23154	130520.466	0.5682	2.5669943
PIL	amoena	1733	1733_16	-84.39869	36.70741	330672.406	0.5623	1.8508317
PIL	deamii	1505	1505_BG17	-87.4839	37.15035	56221.306	0.575	0.7051013

Table S3 Population distances and location (by individual)

garden	taxon	pop	genotype	longitude	latitude	geo_dist	gen_dist	env_dist
PIL	amoena	1727	1727_23	-87.00008	36.23154	130520.466	0.5682	2.5669943
PIL	amoena	1726	1726_03	-88.03654	35.91927	121560.312	0.59236	2.9325467
PIL	pilosa	1718	1718_12	-88.13868	34.62799	265236.246	0.27097	3.6230254
PIL	deamii	1719	1719_01	-87.52286	37.18501	54156.506	0.57941	0.7799909
PIL	pilosa	1714	1714_13	-86.87098	31.78844	592004.221	0.34529	2.6268733
PIL	deamii	1721	1721_12	-88.12802	36.9404	8415.124	0.61006	2.9327668
PIL	amoena	1729	1729_63	-88.0375	35.92486	120934.179	0.60118	2.9325467
PIL	amoena	1733	1733_02	-84.39869	36.70741	330672.406	0.5623	1.8508317
PIL	amoena	1727	1727_17	-87.00008	36.23154	130520.466	0.5682	2.5669943
PIL	amoena	1715	1715_08	-85.65458	33.19064	479624.113	0.55779	2.6120227
PIL	deamii	1721	1721_13	-88.12802	36.9404	8415.124	0.61006	2.9327668
PIL	pilosa	1714	1714_08	-86.87098	31.78844	592004.221	0.34529	2.6268733
PIL	pilosa	1728	1728_24	-88.04331	35.94396	118789.898	0.1798	2.9135217
PIL	pilosa	1728	1728_06	-88.04331	35.94396	118789.898	0.1798	2.9135217
PIL	amoena	1716	1716_13	-87.35549	33.66987	377824.693	0.60086	3.4279444
PIL	amoena	1724	1724_07	-86.80475	36.85496	115853.077	0.55297	0.7101916
PIL	pilosa	1723	1723_09	-87.93854	36.59687	48020.651	0	0.5985479
PIL	pilosa	1729	1729_19	-88.0375	35.92486	120934.179	0.17792	2.9325467
PIL	pilosa	1708	1708_08	-83.52907	30.8033	809149.005	0.45319	4.3437356
PIL	amoena	1715	1715_11	-85.65458	33.19064	479624.113	0.55779	2.6120227
PIL	deamii	1719	1719_13	-87.52286	37.18501	54156.506	0.57941	0.7799909
PIL	amoena	1701	1701_02	-83.14457	34.68221	516105.483	0.59444	2.6902371
PIL	amoena	1726	1726_12	-88.03654	35.91927	121560.312	0.59236	2.9325467
PIL	amoena	1729	1729_45	-88.0375	35.92486	120934.179	0.60118	2.9325467
PIL	amoena	1701	1701_08	-83.14457	34.68221	516105.483	0.59444	2.6902371
PIL	deamii	1720	1720_19	-87.48447	37.14971	56153.158	0.575	0.7051013
PIL	pilosa	1718	1718_02	-88.13868	34.62799	265236.246	0.27097	3.6230254
PIL	amoena	1726	1726_05	-88.03654	35.91927	121560.312	0.59236	2.9325467
PIL	pilosa	1729	1729_15	-88.0375	35.92486	120934.179	0.17792	2.9325467
PIL	pilosa	1712	1712_16	-89.90611	30.91547	698798.812	0.35418	3.9627944
PIL	amoena	1733	1733_09	-84.39869	36.70741	330672.406	0.5623	1.8508317
PIL	pilosa	1713	1713_13	-89.37914	32.21889	546240.51	0.31175	3.904977
PIL	deamii	1720	1720_10	-87.48447	37.14971	56153.158	0.575	0.7051013
PIL	deamii	1720	1720_01	-87.48447	37.14971	56153.158	0.575	0.7051013
PIL	pilosa	1718	1718_16	-88.13868	34.62799	265236.246	0.27097	3.6230254
PIL	pilosa	1712	1712_03	-89.90611	30.91547	698798.812	0.35418	3.9627944
PIL	amoena	1727	1727_01	-87.00008	36.23154	130520.466	0.5682	2.5669943
PIL	pilosa	1713	1713_09	-89.37914	32.21889	546240.51	0.31175	3.904977
PIL	pilosa	1723	1723_10	-87.93854	36.59687	48020.651	0	0.5985479
PIL	amoena	1701	1701_17	-83.14457	34.68221	516105.483	0.59444	2.6902371
PIL	pilosa	1723	1723_02	-87.93854	36.59687	48020.651	0	0.5985479
PIL	pilosa	1718	1718_11	-88.13868	34.62799	265236.246	0.27097	3.6230254
PIL	pilosa	1712	1712_14	-89.90611	30.91547	698798.812	0.35418	3.9627944
PIL	amoena	1715	1715_17	-85.65458	33.19064	479624.113	0.55779	2.6120227
PIL	deamii	1719	1719_19	-87.52286	37.18501	54156.506	0.57941	0.7799909
PIL	pilosa	1728	1728_03	-88.04331	35.94396	118789.898	0.1798	2.9135217
PIL	pilosa	1709	1709_04	-83.42582	30.78985	815434.794	0.45319	4.3437356
PIL	pilosa	1723	1723_12	-87.93854	36.59687	48020.651	0	0.5985479
PIL	deamii	1719	1719_11	-87.52286	37.18501	54156.506	0.57941	0.7799909
PIL	deamii	1719	1719_06	-87.52286	37.18501	54156.506	0.57941	0.7799909
PIL	pilosa	1713	1713_02	-89.37914	32.21889	546240.51	0.31175	3.904977
PIL	amoena	1724	1724_03	-86.80475	36.85496	115853.077	0.55297	0.7101916

Table S3 Population distances and location (by individual)

garden	taxon	pop	genotype	longitude	latitude	geo_dist	gen_dist	env_dist
PIL	deamii	1720	1720_08	-87.48447	37.14971	56153.158	0.575	0.7051013
PIL	amoena	1701	1701_16	-83.14457	34.68221	516105.483	0.59444	2.6902371
PIL	amoena	1716	1716_19	-87.35549	33.66987	377824.693	0.60086	3.4279444
PIL	amoena	1727	1727_11	-87.00008	36.23154	130520.466	0.5682	2.5669943
PIL	amoena	1724	1724_06	-86.80475	36.85496	115853.077	0.55297	0.7101916
PIL	pilosa	1728	1728_01	-88.04331	35.94396	118789.898	0.1798	2.9135217
PIL	pilosa	1704	1704_18	-81.97334	30.77629	894671.612	0.464	5.225737
PIL	deamii	1720	1720_06	-87.48447	37.14971	56153.158	0.575	0.7051013
PIL	pilosa	1714	1714_11	-86.87098	31.78844	592004.221	0.34529	2.6268733
PIL	pilosa	1713	1713_05	-89.37914	32.21889	546240.51	0.31175	3.904977
PIL	deamii	1719	1719_04	-87.52286	37.18501	54156.506	0.57941	0.7799909
PIL	pilosa	1713	1713_08	-89.37914	32.21889	546240.51	0.31175	3.904977
PIL	pilosa	1728	1728_11	-88.04331	35.94396	118789.898	0.1798	2.9135217
PIL	deamii	1719	1719_19	-87.52286	37.18501	54156.506	0.57941	0.7799909
PIL	amoena	1727	1727_15	-87.00008	36.23154	130520.466	0.5682	2.5669943
PIL	amoena	1715	1715_12	-85.65458	33.19064	479624.113	0.55779	2.6120227
PIL	pilosa	1713	1713_17	-89.37914	32.21889	546240.51	0.31175	3.904977
PIL	pilosa	1728	1728_23	-88.04331	35.94396	118789.898	0.1798	2.9135217
PIL	amoena	1701	1701_05	-83.14457	34.68221	516105.483	0.59444	2.6902371
PIL	pilosa	1712	1712_04	-89.90611	30.91547	698798.812	0.35418	3.9627944
PIL	amoena	1729	1729_56	-88.0375	35.92486	120934.179	0.60118	2.9325467
PIL	deamii	1721	1721_13	-88.12802	36.9404	8415.124	0.61006	2.9327668
PIL	amoena	1701	1701_11	-83.14457	34.68221	516105.483	0.59444	2.6902371
PIL	amoena	1726	1726_08	-88.03654	35.91927	121560.312	0.59236	2.9325467
PIL	amoena	1724	1724_13	-86.80475	36.85496	115853.077	0.55297	0.7101916
PIL	pilosa	1729	1729_05	-88.0375	35.92486	120934.179	0.17792	2.9325467
PIL	deamii	1719	1719_12	-87.52286	37.18501	54156.506	0.57941	0.7799909
PIL	deamii	1721	1721_15	-88.12802	36.9404	8415.124	0.61006	2.9327668
PIL	deamii	1721	1721_12	-88.12802	36.9404	8415.124	0.61006	2.9327668
PIL	deamii	1505	1505_BG16	-87.4839	37.15035	56221.306	0.575	0.7051013
PIL	amoena	1716	1716_03	-87.35549	33.66987	377824.693	0.60086	3.4279444
PIL	pilosa	1723	1723_03	-87.93854	36.59687	48020.651	0	0.5985479
PIL	deamii	1720	1720_05	-87.48447	37.14971	56153.158	0.575	0.7051013
PIL	pilosa	1714	1714_05	-86.87098	31.78844	592004.221	0.34529	2.6268733
PIL	amoena	1727	1727_21	-87.00008	36.23154	130520.466	0.5682	2.5669943
PIL	pilosa	1714	1714_14	-86.87098	31.78844	592004.221	0.34529	2.6268733
PIL	pilosa	1729	1729_17	-88.0375	35.92486	120934.179	0.17792	2.9325467
PIL	pilosa	1712	1712_13	-89.90611	30.91547	698798.812	0.35418	3.9627944
PIL	amoena	1729	1729_53	-88.0375	35.92486	120934.179	0.60118	2.9325467
PIL	amoena	1729	1729_61	-88.0375	35.92486	120934.179	0.60118	2.9325467
PIL	amoena	1715	1715_09	-85.65458	33.19064	479624.113	0.55779	2.6120227
PIL	amoena	1727	1727_09	-87.00008	36.23154	130520.466	0.5682	2.5669943
PIL	deamii	1720	1720_17	-87.48447	37.14971	56153.158	0.575	0.7051013
PIL	deamii	1720	1720_08	-87.48447	37.14971	56153.158	0.575	0.7051013
PIL	amoena	1724	1724_16	-86.80475	36.85496	115853.077	0.55297	0.7101916
PIL	deamii	1720	1720_16	-87.48447	37.14971	56153.158	0.575	0.7051013
PIL	pilosa	1723	1723_18	-87.93854	36.59687	48020.651	0	0.5985479
PIL	amoena	1726	1726_02	-88.03654	35.91927	121560.312	0.59236	2.9325467
PIL	pilosa	1712	1712_02	-89.90611	30.91547	698798.812	0.35418	3.9627944
PIL	pilosa	1729	1729_10	-88.0375	35.92486	120934.179	0.17792	2.9325467
PIL	amoena	1729	1729_66	-88.0375	35.92486	120934.179	0.60118	2.9325467
PIL	deamii	1720	1720_20	-87.48447	37.14971	56153.158	0.575	0.7051013

Table S3 Population distances and location (by individual)

garden	taxon	pop	genotype	longitude	latitude	geo_dist	gen_dist	env_dist
PIL	amoena	1716	1716_14	-87.35549	33.66987	377824.693	0.60086	3.4279444
PIL	deamii	1508	1508_BG18	-88.1283	36.9402	8444.601	0.61006	2.9327668
PIL	pilosa	1728	1728_13	-88.04331	35.94396	118789.898	0.1798	2.9135217
PIL	amoena	1726	1726_11	-88.03654	35.91927	121560.312	0.59236	2.9325467
PIL	pilosa	1728	1728_04	-88.04331	35.94396	118789.898	0.1798	2.9135217
PIL	amoena	1726	1726_14	-88.03654	35.91927	121560.312	0.59236	2.9325467
PIL	amoena	1701	1701_20	-83.14457	34.68221	516105.483	0.59444	2.6902371
PIL	deamii	1508	1508_BG18	-88.1283	36.9402	8444.601	0.61006	2.9327668
PIL	pilosa	1723	1723_21	-87.93854	36.59687	48020.651	0	0.5985479
PIL	amoena	1715	1715_02	-85.65458	33.19064	479624.113	0.55779	2.6120227
PIL	amoena	1715	1715_15	-85.65458	33.19064	479624.113	0.55779	2.6120227
PIL	pilosa	1714	1714_01	-86.87098	31.78844	592004.221	0.34529	2.6268733
PIL	pilosa	1704	1704_11	-81.97334	30.77629	894671.612	0.464	5.225737
PIL	pilosa	1718	1718_06	-88.13868	34.62799	265236.246	0.27097	3.6230254
PIL	deamii	1719	1719_18	-87.52286	37.18501	54156.506	0.57941	0.7799909
PIL	amoena	1733	1733_20	-84.39869	36.70741	330672.406	0.5623	1.8508317
PIL	amoena	1733	1733_03	-84.39869	36.70741	330672.406	0.5623	1.8508317
PIL	pilosa	1718	1718_09	-88.13868	34.62799	265236.246	0.27097	3.6230254
PIL	pilosa	1723	1723_07	-87.93854	36.59687	48020.651	0	0.5985479
PIL	pilosa	1718	1718_14	-88.13868	34.62799	265236.246	0.27097	3.6230254
PIL	pilosa	1718	1718_03	-88.13868	34.62799	265236.246	0.27097	3.6230254
PIL	amoena	1716	1716_10	-87.35549	33.66987	377824.693	0.60086	3.4279444
PIL	amoena	1727	1727_03	-87.00008	36.23154	130520.466	0.5682	2.5669943
PIL	pilosa	1714	1714_04	-86.87098	31.78844	592004.221	0.34529	2.6268733
PIL	amoena	1726	1726_09	-88.03654	35.91927	121560.312	0.59236	2.9325467
PIL	amoena	1701	1701_01	-83.14457	34.68221	516105.483	0.59444	2.6902371
PIL	deamii	1720	1720_19	-87.48447	37.14971	56153.158	0.575	0.7051013
PIL	pilosa	1713	1713_07	-89.37914	32.21889	546240.51	0.31175	3.904977
PIL	amoena	1724	1724_18	-86.80475	36.85496	115853.077	0.55297	0.7101916
PIL	pilosa	1718	1718_13	-88.13868	34.62799	265236.246	0.27097	3.6230254
PIL	amoena	1724	1724_08	-86.80475	36.85496	115853.077	0.55297	0.7101916
PIL	amoena	1733	1733_04	-84.39869	36.70741	330672.406	0.5623	1.8508317
PIL	pilosa	1718	1718_08	-88.13868	34.62799	265236.246	0.27097	3.6230254
PIL	pilosa	1709	1709_06	-83.42582	30.78985	815434.794	0.45319	4.3437356
PIL	deamii	1719	1719_08	-87.52286	37.18501	54156.506	0.57941	0.7799909
PIL	amoena	1715	1715_13	-85.65458	33.19064	479624.113	0.55779	2.6120227
PIL	amoena	1716	1716_02	-87.35549	33.66987	377824.693	0.60086	3.4279444
PIL	deamii	1719	1719_16	-87.52286	37.18501	54156.506	0.57941	0.7799909
PIL	deamii	1721	1721_19	-88.12802	36.9404	8415.124	0.61006	2.9327668
PIL	amoena	1727	1727_14	-87.00008	36.23154	130520.466	0.5682	2.5669943
PIL	amoena	1727	1727_12	-87.00008	36.23154	130520.466	0.5682	2.5669943
PIL	pilosa	1713	1713_03	-89.37914	32.21889	546240.51	0.31175	3.904977
PIL	pilosa	1704	1704_10	-81.97334	30.77629	894671.612	0.464	5.225737
PIL	amoena	1727	1727_18	-87.00008	36.23154	130520.466	0.5682	2.5669943
PIL	amoena	1715	1715_07	-85.65458	33.19064	479624.113	0.55779	2.6120227
PIL	amoena	1701	1701_12	-83.14457	34.68221	516105.483	0.59444	2.6902371
PIL	pilosa	1723	1723_17	-87.93854	36.59687	48020.651	0	0.5985479
PIL	pilosa	1728	1728_08	-88.04331	35.94396	118789.898	0.1798	2.9135217
PIL	pilosa	1729	1729_22	-88.0375	35.92486	120934.179	0.17792	2.9325467
PIL	pilosa	1718	1718_07	-88.13868	34.62799	265236.246	0.27097	3.6230254
PIL	amoena	1716	1716_15	-87.35549	33.66987	377824.693	0.60086	3.4279444
PIL	amoena	1724	1724_02	-86.80475	36.85496	115853.077	0.55297	0.7101916

Table S3 Population distances and location (by individual)

garden	taxon	pop	genotype	longitude	latitude	geo_dist	gen_dist	env_dist
PIL	deamii	1720	1720_20	-87.48447	37.14971	56153.158	0.575	0.7051013
PIL	amoena	1715	1715_03	-85.65458	33.19064	479624.113	0.55779	2.6120227
PIL	pilosa	1713	1713_14	-89.37914	32.21889	546240.51	0.31175	3.904977
PIL	pilosa	1729	1729_04	-88.0375	35.92486	120934.179	0.17792	2.9325467
PIL	pilosa	1713	1713_11	-89.37914	32.21889	546240.51	0.31175	3.904977
PIL	pilosa	1728	1728_25	-88.04331	35.94396	118789.898	0.1798	2.9135217
PIL	deamii	1720	1720_01	-87.48447	37.14971	56153.158	0.575	0.7051013
PIL	amoena	1733	1733_15	-84.39869	36.70741	330672.406	0.5623	1.8508317
PIL	deamii	1719	1719_06	-87.52286	37.18501	54156.506	0.57941	0.7799909
PIL	deamii	1720	1720_09	-87.48447	37.14971	56153.158	0.575	0.7051013
PIL	deamii	1720	1720_03	-87.48447	37.14971	56153.158	0.575	0.7051013
PIL	deamii	1719	1719_08	-87.52286	37.18501	54156.506	0.57941	0.7799909
PIL	amoena	1729	1729_64	-88.0375	35.92486	120934.179	0.60118	2.9325467
PIL	pilosa	1712	1712_09	-89.90611	30.91547	698798.812	0.35418	3.9627944
PIL	amoena	1726	1726_21	-88.03654	35.91927	121560.312	0.59236	2.9325467
PIL	deamii	1505	1505_BG16	-87.4839	37.15035	56221.306	0.575	0.7051013
PIL	deamii	1720	1720_16	-87.48447	37.14971	56153.158	0.575	0.7051013
PIL	deamii	1719	1719_18	-87.52286	37.18501	54156.506	0.57941	0.7799909
PIL	deamii	1720	1720_03	-87.48447	37.14971	56153.158	0.575	0.7051013
PIL	amoena	1726	1726_13	-88.03654	35.91927	121560.312	0.59236	2.9325467
PIL	pilosa	1728	1728_12	-88.04331	35.94396	118789.898	0.1798	2.9135217
PIL	amoena	1716	1716_18	-87.35549	33.66987	377824.693	0.60086	3.4279444
PIL	pilosa	1723	1723_11	-87.93854	36.59687	48020.651	0	0.5985479
PIL	deamii	1504	1504_BG15	-87.4961	37.0928	53744.16	0.575	0.7051013
PIL	pilosa	1712	1712_01	-89.90611	30.91547	698798.812	0.35418	3.9627944
PIL	pilosa	1712	1712_12	-89.90611	30.91547	698798.812	0.35418	3.9627944
PIL	pilosa	1723	1723_04	-87.93854	36.59687	48020.651	0	0.5985479
PIL	amoena	1727	1727_07	-87.00008	36.23154	130520.466	0.5682	2.5669943
PIL	amoena	1724	1724_11	-86.80475	36.85496	115853.077	0.55297	0.7101916
PIL	deamii	1721	1721_19	-88.12802	36.9404	8415.124	0.61006	2.9327668
PIL	amoena	1701	1701_04	-83.14457	34.68221	516105.483	0.59444	2.6902371
PIL	pilosa	1728	1728_05	-88.04331	35.94396	118789.898	0.1798	2.9135217
PIL	pilosa	1714	1714_02	-86.87098	31.78844	592004.221	0.34529	2.6268733
PIL	pilosa	1729	1729_16	-88.0375	35.92486	120934.179	0.17792	2.9325467
PIL	amoena	1716	1716_09	-87.35549	33.66987	377824.693	0.60086	3.4279444
PIL	pilosa	1718	1718_20	-88.13868	34.62799	265236.246	0.27097	3.6230254
PIL	pilosa	1704	1704_12	-81.97334	30.77629	894671.612	0.464	5.225737
PIL	amoena	1701	1701_18	-83.14457	34.68221	516105.483	0.59444	2.6902371
PIL	pilosa	1714	1714_15	-86.87098	31.78844	592004.221	0.34529	2.6268733
PIL	amoena	1729	1729_59	-88.0375	35.92486	120934.179	0.60118	2.9325467
PIL	pilosa	1723	1723_05	-87.93854	36.59687	48020.651	0	0.5985479
PIL	deamii	1720	1720_09	-87.48447	37.14971	56153.158	0.575	0.7051013
PIL	pilosa	1723	1723_22	-87.93854	36.59687	48020.651	0	0.5985479
PIL	pilosa	1718	1718_01	-88.13868	34.62799	265236.246	0.27097	3.6230254
PIL	amoena	1729	1729_57	-88.0375	35.92486	120934.179	0.60118	2.9325467
PIL	amoena	1726	1726_01	-88.03654	35.91927	121560.312	0.59236	2.9325467
PIL	pilosa	1714	1714_09	-86.87098	31.78844	592004.221	0.34529	2.6268733
PIL	pilosa	1729	1729_12	-88.0375	35.92486	120934.179	0.17792	2.9325467
PIL	deamii	1720	1720_14	-87.48447	37.14971	56153.158	0.575	0.7051013
PIL	amoena	1729	1729_48	-88.0375	35.92486	120934.179	0.60118	2.9325467
PIL	amoena	1715	1715_14	-85.65458	33.19064	479624.113	0.55779	2.6120227
PIL	deamii	1721	1721_22	-88.12802	36.9404	8415.124	0.61006	2.9327668

Table S3 Population distances and location (by individual)

garden	taxon	pop	genotype	longitude	latitude	geo_dist	gen_dist	env_dist
PIL	deamii	1720	1720_14	-87.48447	37.14971	56153.158	0.575	0.7051013
PIL	pilosa	1704	1704_09	-81.97334	30.77629	894671.612	0.464	5.225737
PIL	amoena	1715	1715_06	-85.65458	33.19064	479624.113	0.55779	2.6120227
PIL	amoena	1716	1716_07	-87.35549	33.66987	377824.693	0.60086	3.4279444
PIL	deamii	1720	1720_06	-87.48447	37.14971	56153.158	0.575	0.7051013
PIL	deamii	1721	1721_21	-88.12802	36.9404	8415.124	0.61006	2.9327668
PIL	pilosa	1708	1708_01	-83.52907	30.8033	809149.005	0.45319	4.3437356
PIL	amoena	1716	1716_04	-87.35549	33.66987	377824.693	0.60086	3.4279444
PIL	amoena	1727	1727_08	-87.00008	36.23154	130520.466	0.5682	2.5669943
PIL	deamii	1719	1719_04	-87.52286	37.18501	54156.506	0.57941	0.7799909
PIL	pilosa	1729	1729_11	-88.0375	35.92486	120934.179	0.17792	2.9325467
PIL	pilosa	1714	1714_10	-86.87098	31.78844	592004.221	0.34529	2.6268733
PIL	deamii	1719	1719_20	-87.52286	37.18501	54156.506	0.57941	0.7799909
PIL	pilosa	1713	1713_19	-89.37914	32.21889	546240.51	0.31175	3.904977
PIL	amoena	1726	1726_07	-88.03654	35.91927	121560.312	0.59236	2.9325467
PIL	deamii	1719	1719_20	-87.52286	37.18501	54156.506	0.57941	0.7799909
PIL	pilosa	1723	1723_16	-87.93854	36.59687	48020.651	0	0.5985479
PIL	amoena	1724	1724_17	-86.80475	36.85496	115853.077	0.55297	0.7101916
PIL	amoena	1715	1715_10	-85.65458	33.19064	479624.113	0.55779	2.6120227
PIL	amoena	1729	1729_54	-88.0375	35.92486	120934.179	0.60118	2.9325467
PIL	pilosa	1723	1723_08	-87.93854	36.59687	48020.651	0	0.5985479
PIL	deamii	1504	1504_BG15	-87.4961	37.0928	53744.16	0.575	0.7051013
PIL	deamii	1603	1603_BG56	-87.4962	37.09251	53730.426	0.575	0.7051013
PIL	deamii	1721	1721_22	-88.12802	36.9404	8415.124	0.61006	2.9327668
PIL	amoena	1701	1701_13	-83.14457	34.68221	516105.483	0.59444	2.6902371
PIL	pilosa	1713	1713_04	-89.37914	32.21889	546240.51	0.31175	3.904977
PIL	amoena	1715	1715_05	-85.65458	33.19064	479624.113	0.55779	2.6120227
PIL	amoena	1724	1724_04	-86.80475	36.85496	115853.077	0.55297	0.7101916
PIL	amoena	1701	1701_07	-83.14457	34.68221	516105.483	0.59444	2.6902371
PIL	deamii	1603	1603_BG56	-87.4962	37.09251	53730.426	0.575	0.7051013
PIL	pilosa	1712	1712_05	-89.90611	30.91547	698798.812	0.35418	3.9627944
PIL	pilosa	1713	1713_15	-89.37914	32.21889	546240.51	0.31175	3.904977
PIL	amoena	1727	1727_16	-87.00008	36.23154	130520.466	0.5682	2.5669943
PIL	pilosa	1728	1728_17	-88.04331	35.94396	118789.898	0.1798	2.9135217
PIL	deamii	1720	1720_05	-87.48447	37.14971	56153.158	0.575	0.7051013
PIL	pilosa	1729	1729_02	-88.0375	35.92486	120934.179	0.17792	2.9325467
PIL	pilosa	1718	1718_04	-88.13868	34.62799	265236.246	0.27097	3.6230254
PIL	pilosa	1728	1728_02	-88.04331	35.94396	118789.898	0.1798	2.9135217
PIL	pilosa	1709	1709_08	-83.42582	30.78985	815434.794	0.45319	4.3437356
PIL	deamii	1721	1721_09	-88.12802	36.9404	8415.124	0.61006	2.9327668
PIL	amoena	1724	1724_10	-86.80475	36.85496	115853.077	0.55297	0.7101916
PIL	deamii	1720	1720_02	-87.48447	37.14971	56153.158	0.575	0.7051013
PIL	pilosa	1718	1718_15	-88.13868	34.62799	265236.246	0.27097	3.6230254
PIL	amoena	1727	1727_10	-87.00008	36.23154	130520.466	0.5682	2.5669943
PIL	pilosa	1723	1723_19	-87.93854	36.59687	48020.651	0	0.5985479
PIL	pilosa	1714	1714_18	-86.87098	31.78844	592004.221	0.34529	2.6268733
PIL	pilosa	1728	1728_07	-88.04331	35.94396	118789.898	0.1798	2.9135217
PIL	amoena	1715	1715_16	-85.65458	33.19064	479624.113	0.55779	2.6120227
PIL	pilosa	1714	1714_07	-86.87098	31.78844	592004.221	0.34529	2.6268733
PIL	pilosa	1712	1712_15	-89.90611	30.91547	698798.812	0.35418	3.9627944
PIL	deamii	1719	1719_12	-87.52286	37.18501	54156.506	0.57941	0.7799909
PIL	deamii	1719	1719_11	-87.52286	37.18501	54156.506	0.57941	0.7799909

Table S3 Population distances and location (by individual)

**garden**	**taxon**	**pop**	**genotype**	**longitude**	**latitude**	**geo_dist**	**gen_dist**	**env_dist**
PIL	pilosa	1718	1718_17	-88.13868	34.62799	265236.246	0.27097	3.6230254
PIL	deamii	1720	1720_17	-87.48447	37.14971	56153.158	0.575	0.7051013
PIL	pilosa	1713	1713_10	-89.37914	32.21889	546240.51	0.31175	3.904977
PIL	amoena	1726	1726_19	-88.03654	35.91927	121560.312	0.59236	2.9325467
PIL	amoena	1733	1733_10	-84.39869	36.70741	330672.406	0.5623	1.8508317
PIL	amoena	1726	1726_04	-88.03654	35.91927	121560.312	0.59236	2.9325467
PIL	pilosa	1723	1723_13	-87.93854	36.59687	48020.651	0	0.5985479
PIL	amoena	1701	1701_10	-83.14457	34.68221	516105.483	0.59444	2.6902371
PIL	deamii	1720	1720_07	-87.48447	37.14971	56153.158	0.575	0.7051013
PIL	amoena	1733	1733_11	-84.39869	36.70741	330672.406	0.5623	1.8508317
PIL	pilosa	1714	1714_19	-86.87098	31.78844	592004.221	0.34529	2.6268733
PIL	pilosa	1713	1713_01	-89.37914	32.21889	546240.51	0.31175	3.904977
PIL	amoena	1716	1716_17	-87.35549	33.66987	377824.693	0.60086	3.4279444
PIL	pilosa	1718	1718_10	-88.13868	34.62799	265236.246	0.27097	3.6230254
PIL	deamii	1721	1721_09	-88.12802	36.9404	8415.124	0.61006	2.9327668
PIL	amoena	1729	1729_62	-88.0375	35.92486	120934.179	0.60118	2.9325467
PIL	amoena	1729	1729_65	-88.0375	35.92486	120934.179	0.60118	2.9325467
PIL	amoena	1727	1727_02	-87.00008	36.23154	130520.466	0.5682	2.5669943
PIL	pilosa	1709	1709_12	-83.42582	30.78985	815434.794	0.45319	4.3437356
PIL	pilosa	1729	1729_01	-88.0375	35.92486	120934.179	0.17792	2.9325467
PIL	amoena	1727	1727_20	-87.00008	36.23154	130520.466	0.5682	2.5669943
PIL	amoena	1716	1716_11	-87.35549	33.66987	377824.693	0.60086	3.4279444
PIL	pilosa	1729	1729_20	-88.0375	35.92486	120934.179	0.17792	2.9325467
PIL	amoena	1724	1724_09	-86.80475	36.85496	115853.077	0.55297	0.7101916
PIL	amoena	1726	1726_10	-88.03654	35.91927	121560.312	0.59236	2.9325467
PIL	amoena	1729	1729_49	-88.0375	35.92486	120934.179	0.60118	2.9325467
PIL	pilosa	1723	1723_01	-87.93854	36.59687	48020.651	0	0.5985479

Table S4 Distance by fitness regression model results without adjustement for multiple testing.

garden	taxon	trait	distance	intercep	coefficient	Rsquared	pvalue
PIL	pilosa	biomass	geo_dist	7.75	-0.0010	0.5597	**0.02043**
PIL	pilosa	biomass	gen_dist	7.81	-1.7547	0.3910	0.07176
PIL	pilosa	biomass	env2_dist	7.97	-0.1972	0.3906	0.07194
PIL	pilosa	flowers #	geo_dist	4.70	-0.0028	0.5838	**0.01653**
PIL	pilosa	flowers #	gen_dist	5.05	-5.7331	0.5094	**0.03082**
PIL	pilosa	flowers #	env2_dist	5.56	-0.6399	0.5021	**0.03261**
PIL	pilosa	fruit #	geo_dist	4.08	-0.0015	0.6332	**0.01032**
PIL	pilosa	fruit #	gen_dist	4.29	-3.0900	0.5927	**0.01524**
PIL	pilosa	fruit #	env2_dist	4.57	-0.3460	0.5868	**0.01608**
PIL	pilosa	herbivory	geo_dist	0.49	0.0009	0.3337	0.1033
PIL	pilosa	herbivory	gen_dist	0.41	1.6782	0.2683	0.1531
PIL	pilosa	herbivory	env2_dist	0.47	0.1229	0.1139	0.3745
PIL	pilosa	survival	geo_dist	-0.70	0.0000	0.0123	0.7765
PIL	pilosa	survival	env2_dist	-0.70	0.0000	0.0098	0.8002
PIL	pilosa	survival	gen_dist	-0.70	0.0000	0.0074	0.826
PIL	amoena	biomass	geo_dist	7.56	-0.0015	0.3501	0.1223
PIL	amoena	biomass	gen_dist	7.36	-0.3763	0.0003	0.9678
PIL	amoena	biomass	env2_dist	7.16	-0.0083	0.0002	0.9709
PIL	amoena	flowers #	geo_dist	4.00	-0.0020	0.2975	0.1621
PIL	amoena	flowers #	env2_dist	3.28	0.0672	0.0078	0.8357
PIL	amoena	flowers #	gen_dist	4.64	-2.0620	0.0043	0.8769
PIL	amoena	fruit #	geo_dist	3.40	-0.0006	0.1275	0.3852
PIL	amoena	fruit #	env2_dist	3.00	0.0931	0.0657	0.5399
PIL	amoena	fruit #	gen_dist	1.35	3.2400	0.0473	0.6049
PIL	amoena	herbivory	env2_dist	2.28	0.0000	0.8832	**0.000521**
PIL	amoena	herbivory	gen_dist	2.28	0.0000	0.3734	0.1075
PIL	amoena	herbivory	geo_dist	2.28	0.0000	0.0819	0.492
PIL	amoena	survival	env2_dist	-0.96	0.0937	0.2673	0.1895
PIL	amoena	survival	gen_dist	-2.11	2.3970	0.1039	0.4362
PIL	amoena	survival	geo_dist	-0.66	-0.0002	0.0761	0.5083
DEA	pilosa	biomass	gen_dist	15.37	-13.6730	0.5517	**0.02187**
DEA	pilosa	biomass	geo_dist	7.69	-0.0011	0.3218	0.1112
DEA	pilosa	biomass	env2_dist	8.00	-0.2336	0.2574	0.1632
DEA	pilosa	flowers #	gen_dist	24.43	-35.8320	0.6850	**0.005887**
DEA	pilosa	flowers #	geo_dist	4.41	-0.0032	0.4647	**0.04313**
DEA	pilosa	flowers #	env2_dist	4.96	-0.5733	0.2802	0.1428
DEA	pilosa	fruit #	gen_dist	27.47	-40.3500	0.6917	**0.005444**
DEA	pilosa	fruit #	geo_dist	4.85	-0.0034	0.4309	0.05481
DEA	pilosa	fruit #	env2_dist	5.92	-0.7508	0.3827	0.07573
DEA	pilosa	herbivory	gen_dist	-4.84	9.4220	0.3373	0.101
DEA	pilosa	herbivory	env2_dist	0.01	0.2270	0.3133	0.1171
DEA	pilosa	herbivory	geo_dist	0.52	0.0006	0.1306	0.3393
DEA	pilosa	survival	env2_dist	-0.70	0.0000	0.1642	0.2793
DEA	pilosa	survival	gen_dist	-0.70	0.0000	0.1090	0.3855

Table S4 Distance by fitness regression model results without adjustement for multiple testing.

garden	taxon	trait	distance	intercep	coefficient	Rsquared	pvalue
DEA	pilosa	survival	geo_dist	-0.70	0.0000	0.0721	0.4849
DEA	amoena	biomass	geo_dist	7.46	-0.0006	0.1285	0.3832
DEA	amoena	biomass	env2_dist	7.53	-0.0905	0.0757	0.5097
DEA	amoena	biomass	gen_dist	7.35	-0.0919	0.0000	0.9869
DEA	amoena	flowers #	geo_dist	3.82	-0.0020	0.3251	0.14
DEA	amoena	flowers #	env2_dist	3.99	-0.2780	0.1689	0.3118
DEA	amoena	flowers #	gen_dist	9.55	-11.1290	0.1686	0.3123
DEA	amoena	fruit #	geo_dist	3.39	-0.0008	0.3060	0.155
DEA	amoena	fruit #	env2_dist	3.46	-0.1139	0.1623	0.3223
DEA	amoena	fruit #	gen_dist	4.67	-2.6600	0.0551	0.5759
DEA	amoena	herbivory	geo_dist	2.28	0.0000	0.1721	0.3068
DEA	amoena	herbivory	gen_dist	2.28	0.0000	0.0912	0.4672
DEA	amoena	herbivory	env2_dist	2.28	0.0000	0.0019	0.9187
DEA	amoena	survival	gen_dist	2.05	-4.9720	0.5420	**0.0373**
DEA	amoena	survival	env2_dist	-0.44	-0.1210	0.5162	**0.04466**
DEA	amoena	survival	geo_dist	-0.60	-0.0005	0.3808	0.1031
AMO	pilosa	biomass	gen_dist	12.56	-9.1470	0.6332	**0.01032**
AMO	pilosa	biomass	geo_dist	7.60	-0.0012	0.5174	**0.02894**
AMO	pilosa	biomass	env2_dist	7.75	-0.4198	0.4352	0.05318
AMO	pilosa	flowers #	gen_dist	18.34	-26.1590	0.7691	**0.001902**
AMO	pilosa	flowers #	geo_dist	4.04	-0.0030	0.5209	**0.02815**
AMO	pilosa	flowers #	env2_dist	4.53	-1.1575	0.4915	**0.03537**
AMO	pilosa	fruit #	gen_dist	13.84	-18.1130	0.5354	**0.02503**
AMO	pilosa	fruit #	env2_dist	4.42	-0.8950	0.4267	0.05644
AMO	pilosa	fruit #	geo_dist	3.95	-0.0021	0.3699	0.08227
AMO	pilosa	herbivory	gen_dist	-13.97	24.7850	0.8128	**0.000894**
AMO	pilosa	herbivory	geo_dist	-0.52	0.0031	0.6487	**0.008792**
AMO	pilosa	herbivory	env2_dist	-0.75	1.0007	0.4324	0.05423
AMO	pilosa	survival	geo_dist	-0.70	0.0000	0.0296	0.6579
AMO	pilosa	survival	gen_dist	-0.70	0.0000	0.0275	0.6699
AMO	pilosa	survival	env2_dist	-0.70	0.0000	0.0054	0.851
AMO	amoena	biomass	env2_dist	7.05	0.0717	0.0596	0.5602
AMO	amoena	biomass	geo_dist	7.20	-0.0003	0.0314	0.6746
AMO	amoena	biomass	gen_dist	7.12	0.0384	0.0004	0.9645
AMO	amoena	flowers #	env2_dist	3.33	-0.1497	0.0258	0.7038
AMO	amoena	flowers #	geo_dist	3.35	-0.0009	0.0218	0.7273
AMO	amoena	flowers #	gen_dist	3.30	-0.4489	0.0049	0.8694
AMO	amoena	fruit #	geo_dist	3.29	-0.0008	0.0634	0.5473
AMO	amoena	fruit #	env2_dist	3.04	0.0964	0.0393	0.6378
AMO	amoena	fruit #	gen_dist	3.12	0.0816	0.0006	0.9544
AMO	amoena	herbivory	env2_dist	2.28	0.0000	0.3158	0.1472
AMO	amoena	herbivory	geo_dist	2.28	0.0000	0.1236	0.393
AMO	amoena	herbivory	gen_dist	2.28	0.0000	0.0252	0.7072
AMO	amoena	survival	gen_dist	-0.55	-0.3810	0.1391	0.3628

Table S4 Distance by fitness regression model results without adjustement for multiple testing.

garden	taxon	trait	distance	intercep	coefficient	Rsquared	pvalue
AMO	amoena	survival	geo_dist	-0.63	-0.0001	0.0215	0.7293
AMO	amoena	survival	env2_dist	-0.64	-0.0129	0.0076	0.8376

Table S5 Source Populations

population	taxon	longitude	latitude	Number of Genotypes
Amo garden	amoena	-87.000084	36.231544	NA
Dea garden	deamii	-87.484469	37.149707	NA
Pil garden	pilosa	-88.092145	37.010345	NA
1701	amoena	-83.144574	34.682213	15
1715	amoena	-85.654577	33.190636	16
1716	amoena	-87.355493	33.669868	16
1724	amoena	-86.80475	36.85496	16
1726	amoena	-88.036543	35.919266	16
1727	amoena	-87.000084	36.231544	19
1729	amoena	-88.037497	35.924864	15
1729	amoena	-88.037497	35.924864	14
1733	amoena	-84.398694	36.707411	9
1719	deamii	-87.522865	37.185007	11
1720	deamii	-87.484469	37.149707	18
1721	deamii	-88.128025	36.940396	8
1704	pilosa	-81.973344	30.776288	6
1708	pilosa	-83.529067	30.803303	7
1712	pilosa	-89.906109	30.915467	12
1713	pilosa	-89.379136	32.218893	16
1714	pilosa	-86.870979	31.788445	16
1718	pilosa	-88.138683	34.627986	18
1723	pilosa	-87.938538	36.596873	20
1728	pilosa	-88.043306	35.94396	16

Table S6 Garden Sites

Longitude	Latitude	Habitat	Description
-88.092145	37.010345	Phlox pilosa subsp. pilosa	flat, grass-dominated meadow with a community of interspersed herbaceous flowering plants, 50 meters from a mixed hardwood forest
-87.48399	37.15035	Phlox pilosa subsp. deamii	steeply sloped, partially wooded bank supporting a diverse community of herbaceous plants and shrubs at the edge of an oak-hickory forest
-87.000084	36.231544	Phlox amoena subsp. amoena	rocky, gently sloping roadside supporting a diverse community of herbaceous plants, adjacent to a mixed hardwood forest (oak, hickory, dogwood, maple, ash)

Table S7 Summary of leaf trait PCA with proportion and cummulative variance explained by first three PC axes and trait loadings on first three PCs (E Matrix from Chong et al 2018)

	PC1	PC2	PC3
Total Proportion Variance Explained	0.450	0.278	0.182
Cummulative Variance Explained	0.450	0.728	0.910
**TRAIT LOADINGS**			
length	0.519	0.382	0.002
width	-0.439	0.430	-0.347
area	0.135	0.715	-0.276
length/width ratio	0.583	-0.007	0.199
chlorophyl content	-0.418	0.181	0.414
Specific leaf area	0.076	-0.354	-0.769

Chong, V.K. Fung, H.F & Stinchcombe, J.R. A note on measuring natural selection on principal component scores. Evol Lett 2, 272-280 (2018). https//doi.org:10.1002/evl3.63

Table S8 Linear model results of how fitness traits are predicted by first two PCs of leaf trait variation while controlling for species identity with bolded results indicating significance at $p<0.05$ uncorrected for multiple tests.

	**Leaf Trait PC1**						**Leaf Trait PC2**					
	**Trait PC1**		**Taxon**		**Taxon:PC1**		**Trait PC2**		**Taxon**		**Taxon:PC2**	
	F value	P value	F value	P value	F value	P value	F value	P value	F value	P value	F value	P value
**Fruit**	1.10	0.295	**28.43**	**<0.001**	0.13	0.877	3.63	0.058	93.01	<0.001	**5.48**	**0.005**
**Flower**	0.06	0.804	**5.31**	**0.005**	0.11	0.896	5.40	<0.001	16.83	<0.001	**3.27**	**0.039**
**Biomass**	0.70	0.402	**12.61**	**0.014**	0.35	0.702	11.51	<0.001	25.79	<0.001	**3.94**	**0.02**

Table S9 Linear model results of how PC1 and PC2 predict fitness traits for all species combined and within each species with bolded results indicating significance at $p<0.05$.

	**Leaf Trait PC1**											
	**combined**			**pilosa**			**amoena**			**deamii**		
	coef	std err	P value	coef	std err	P value	coef	std err	P value	coef	std err	P value
**Fruit**	**0.644**	**0.068**	**<0.001**	-0.184	0.249	0.46	0.069	0.097	0.475	-0.044	0.143	0.76
**Flower**	**0.251**	**0.063**	**<0.001**	-0.012	0.21	0.58	-0.049	0.155	0.751	0.044	0.232	0.85
**Biomass**	**0.265**	**0.042**	**<0.001**	0.008	0.15	0.959	0.157	0.09	0.083	0.071	0.17	0.678

	**Leaf Trait PC2**											
	**combined**			**pilosa**			**amoena**			**deamii**		
	coef	std err	P value	coef	std err	P value	coef	std err	P value	coef	std err	P value
**Fruit**	**0.323**	**0.097**	**<0.001**	**0.61**	**0.157**	**0.001**	**0.251**	**0.062**	**<0.001**	-0.108	0.099	0.276
**Flower**	**0.223**	**0.081**	**0.006**	**0.346**	**0.136**	**0.012**	**0.3067**	**0.102**	**0.003**	-0.254	0.158	0.112
**Biomass**	**0.153**	**0.057**	**0.008**	**0.286**	**0.097**	**0.0037**	**0.146**	**0.06**	**0.0168**	-0.17	0.116	0.148

Table S10 Summary of all regression coefficents of fitness measures and first three PCs of leaf traits for all species and just Pilosa data (A vectors from Chong et al 2018)

	All species			Pilosa only		
	PC1	PC2	PC3	PC1	PC2	PC3
Flower number	0.251	0.223	0.081	-0.116	0.346	0.281
Fruit number	0.644	0.323	0.328	-0.184	0.610	0.430
Biomass	0.265	0.153	0.058	0.008	0.286	0.162

Chong, V.K. Fung, H.F & Stinchcombe, J.R. A note on measuring natural selection on principal component scores. Evol Lett 2, 272-280 (2018). https//doi.org:10.1002/evl3.63

Table S11:Results from ANOVA model comparing leaf traits between field and greenhouse grown plants.

	**Taxon**			**Location**			**Taxon:Location**		
	Sum Sq	F	p	Sum Sq	F	p	Sum Sq	F	p
Length:Width Ratio	1698.70	225.00	<0.001	4.50	0.60	0.442	21.03	2.79	0.098
Leaf Length	138.27	132.32	<0.001	38.89	37.22	<0.001	0.04	0.04	0.837
Leaf area	9.69	20.29	<0.001	35.22	73.75	<0.001	1.09	2.28	0.135
Leaf Width	0.40	60.17	<0.001	0.80	120.30	<0.001	0.04	5.76	0.018
Specific Leaf Area	929.00	0.54	0.464	4825.00	2.81	0.097	9398.00	5.48	0.021

Taxon include amoena and pilosa and location includes greenhouse and field measure traits.
