# Supplementary material for: A multidimensional selective landscape drives adaptive divergence between and within closely related Phlox species
Source: Nat Commun. 2024 May 31;15:4661. doi: 10.1038/s41467-024-49075-6 (PMC11143288; doi:10.1038/s41467-024-49075-6)
Supplement: Supplementary file 3 — Reporting Summary [file 41467_2024_49075_MOESM3_ESM.pdf]

nature portfolio

Corresponding author(s): Robin Hopkins

Last updated by author(s): April 15, 2024

Reporting Summary

Nature Portfolio wishes to improve the reproducibility of the work that we publish. This form provides structure for consistency and transparency in reporting. For further information on Nature Portfolio policies, see our Editorial Policies and the Editorial Policy Checklist.

Please do not complete any field with "not applicable" or n/a. Refer to the help text for what text to use if an item is not relevant to your study. For final submission: please carefully check your responses for accuracy; you will not be able to make changes later.

Statistics

For all statistical analyses, confirm that the following items are present in the figure legend, table legend, main text, or Methods section.

n/a	Confirmed	
☐	☑	The exact sample size (*n*) for each experimental group/condition, given as a discrete number and unit of measurement
☐	☑	A statement on whether measurements were taken from distinct samples or whether the same sample was measured repeatedly
☐	☑	The statistical test(s) used AND whether they are one- or two-sided<br>*Only common tests should be described solely by name; describe more complex techniques in the Methods section.*
☐	☑	A description of all covariates tested
☐	☑	A description of any assumptions or corrections, such as tests of normality and adjustment for multiple comparisons
☐	☑	A full description of the statistical parameters including central tendency (e.g. means) or other basic estimates (e.g. regression coefficient) AND variation (e.g. standard deviation) or associated estimates of uncertainty (e.g. confidence intervals)
☐	☑	For null hypothesis testing, the test statistic (e.g. *F*, *t*, *r*) with confidence intervals, effect sizes, degrees of freedom and *P* value noted<br>*Give P values as exact values whenever suitable.*
☐	☑	For Bayesian analysis, information on the choice of priors and Markov chain Monte Carlo settings
☐	☑	For hierarchical and complex designs, identification of the appropriate level for tests and full reporting of outcomes
☐	☑	Estimates of effect sizes (e.g. Cohen's *d*, Pearson's *r*), indicating how they were calculated

Our web collection on statistics for biologists contains articles on many of the points above.

Software and code

Policy information about availability of computer code

Data collection	No software was used for data collection.
Data analysis	R version 4.2.3 was used to analyze data. All code generated for this project is availible at: https://github.com/PhloxHopkins/PhloxFieldAdaptation

For manuscripts utilizing custom algorithms or software that are central to the research but not yet described in published literature, software must be made available to editors and reviewers. We strongly encourage code deposition in a community repository (e.g. GitHub). See the Nature Portfolio guidelines for submitting code & software for further information.

Data

Policy information about availability of data

All manuscripts must include a data availability statement. This statement should provide the following information, where applicable:

- Accession codes, unique identifiers, or web links for publicly available datasets
- A description of any restrictions on data availability
- For clinical datasets or third party data, please ensure that the statement adheres to our policy

Data are availible on Dryad at DOI:dryad.gxd2547.sx . We included occurrence data from the Global Biodiversity Information Facility (GBIF; https://www.gbif.org/) and the Southeast Regional Network of Expertise and Collections (SERNEC; https:// sernecportal.org/portal/). We extracted bioclimatic variables from the WorldClim dataset (https://www.worldclim.org/data/bioclim.html) and soil composition and chemistry variables from the Unified North America Soil Map (https://daac.ornl.gov/NACP/guides/NACP MsTMIP Unified_NA_Soil_Map.html)

Research involving human participants, their data, or biological material

Policy information about studies with human participants or human data. See also policy information about sex, gender (identity/presentation), and sexual orientation and race, ethnicity and racism.

Reporting on sex and gender	NA
Reporting on race, ethnicity, or other socially relevant groupings	NA
Population characteristics	NA
Recruitment	NA
Ethics oversight	NA

Note that full information on the approval of the study protocol must also be provided in the manuscript.

Field-specific reporting

Please select the one below that is the best fit for your research. If you are not sure, read the appropriate sections before making your selection.

☐ Life sciences ☐ Behavioural & social sciences ☑ Ecological, evolutionary & environmental sciences

For a reference copy of the document with all sections, see nature.com/documents/nr-reporting-summary-flat.pdf

Life sciences study design

All studies must disclose on these points even when the disclosure is negative.

Sample size	
Data exclusions	
Replication	
Randomization	
Blinding	

Behavioural & social sciences study design

All studies must disclose on these points even when the disclosure is negative.

Study description	
Research sample	
Sampling strategy	
Data collection	
Timing	
Data exclusions	
Non-participation	
Randomization	

Ecological, evolutionary & environmental sciences study design

All studies must disclose on these points even when the disclosure is negative.

Study description	This work includes three common gardens each containing 321 individuals of three Phlox species that are clonally replicated across gardens. The data include measurements of leaves, flowers, fruits, survival across three years in natural environmental conditions. All individuals were randomized in location within each gardens.
Research sample	The plants included in this study include 122 individuals of Phlox amoena (from 8 populations), 125 individuals of Phlox pilosa (from 9 populations), and 37 genotypes of Phlox pilosa deamii (from 3 populations). These populations are sampled from the across the ranges of these three species and the diversity includes the known diversity of these species.
Sampling strategy	Ten to twenty individuals from populations across the species ranges were chosen to be included in this experiment. The number of populations used were limited by availability of found populations, the number of individuals per population was limited by ability and availability of propagating individuals in the greenhouse. Total sample size was limited by human feasibility and space limitations.
Data collection	Data were collected by the authors over the course of three field seasons. All data were collected with pencil and paper and entered into electronic data files. Data were collected without knowledge of species identity or population source at time of collection.
Timing and spatial scale	Common gardens that were approximately 25X25 meters were planted in April 2018 and data collection was completed in September 2020. Data was collected at non-regular time intervals throughout the growing season of 2018 and 2019. Visits to the field sites in 2020 was limited due to the COVID pandemic. We were only able to collect final survival in September of 2020.
Data exclusions	We included all available and verifiable data in our analyses.
Reproducibility	This experiment was performed one time across three spatial replicates and over a period of three years. It was not replicated in its entirety. We repeated measurements of leaf traits on plants grown in the field and in the greenhouse to verify that the differences between species were robust and reproducible across environments.
Randomization	All gardens included clonal replicates of the same individuals and all individuals were randomly positions across blocks at each garden site. Each block contained the same number of individuals from each species but in a different randomized order.
Blinding	Experimental plants were labeled with anonymous numbers and data was collected by number without knowledge of the plant species or source location.

Did the study involve field work? ☑ Yes ☐ No

Field work, collection and transport

Field conditions	The field work took place over three years with variable conditions and weather.
Location	The common gardens were located at (-88.0921, 37.0103) & (-87.484, 37.150) & (-87.000, 36.2315).
Access & import/export	All samples included in this study were collected with permits from the US forest service (USDA #005277 05/2017) and the South Carolina Department of Natural resources (SCDNR SU-12-2017 04/2017) and/or with permission from private property owners. The use of the land at Land Between the Lakes was used in accordance with permit #LBL17170.
Disturbance	The garden sites were minimally disturbed with plantings and all plants and materials were removed at end of experiment. Fruits and seeds were collected to the best of our abilities as plants matured.

Reporting for specific materials, systems and methods

We require information from authors about some types of materials, experimental systems and methods used in many studies. Here, indicate whether each material, system or method listed is relevant to your study. If you are not sure if a list item applies to your research, read the appropriate section before selecting a response.

Materials & experimental systems

n/a	Involved in the study	
x	☐	Antibodies
x	☐	Eukaryotic cell lines
x	☐	Palaeontology and archaeology
x	☐	Animals and other organisms
x	☐	Clinical data
x	☐	Dual use research of concern
☐	☑	Plants

Methods

n/a	Involved in the study	
x	☐	ChIP-seq
x	☐	Flow cytometry
x	☐	MRI-based neuroimaging

Antibodies

Antibodies used	NA
Validation	NA

Eukaryotic cell lines

Policy information about cell lines and Sex and Gender in Research

Cell line source(s)	NA
Authentication	NA
Mycoplasma contamination	NA
Commonly misidentified lines (See ICLAC register)	NA

Palaeontology and Archaeology

Specimen provenance	NA
Specimen deposition	NA
Dating methods	NA

☐ Tick this box to confirm that the raw and calibrated dates are available in the paper or in Supplementary Information.

Ethics oversight	NA

Note that full information on the approval of the study protocol must also be provided in the manuscript.

Animals and other research organisms

Policy information about studies involving animals; ARRIVE guidelines recommended for reporting animal research, and Sex and Gender in Research

Laboratory animals	NA
Wild animals	NA
Reporting on sex	NA
Field-collected samples	NA
Ethics oversight	NA

Note that full information on the approval of the study protocol must also be provided in the manuscript.

Clinical data

Policy information about clinical studies
All manuscripts should comply with the ICMJE guidelines for publication of clinical research and a completed CONSORT checklist must be included with all submissions.

Clinical trial registration	
Study protocol	
Data collection	
Outcomes	

Dual use research of concern

Policy information about dual use research of concern

Hazards

Could the accidental, deliberate or reckless misuse of agents or technologies generated in the work, or the application of information presented in the manuscript, pose a threat to:

No	Yes	
[x]	[ ]	Public health
[x]	[ ]	National security
[x]	[ ]	Crops and/or livestock
[x]	[ ]	Ecosystems
[x]	[ ]	Any other significant area

Experiments of concern

Does the work involve any of these experiments of concern:

No	Yes	
[x]	[ ]	Demonstrate how to render a vaccine ineffective
[x]	[ ]	Confer resistance to therapeutically useful antibiotics or antiviral agents
[x]	[ ]	Enhance the virulence of a pathogen or render a nonpathogen virulent
[x]	[ ]	Increase transmissibility of a pathogen
[x]	[ ]	Alter the host range of a pathogen
[x]	[ ]	Enable evasion of diagnostic/detection modalities
[x]	[ ]	Enable the weaponization of a biological agent or toxin
[x]	[ ]	Any other potentially harmful combination of experiments and agents

Plants

Seed stocks

Plants were collected from 20 natural populations and GPS coordinates are provided in Table S5.
-87.000084, 36.231544; -87.484469, 37.149707 ; -88.092145, 37.010345; -83.14457, 34.682213; -85.654577, 33.190636; -87.355493,33.669868; -86.80475, 36.85496; -88.036543, 35.919266; -87.000084 , 36.231544; -88.037497,35.924864; -88.037497, 35.924864; -84.398694, 36.707411 ; -87.522865, 37.185007; -87.484469, 37.149707; -88.128025, 36.940396; -81.973344, 30.776288; -83.529067, 30.803303; -89.906109, 30.915467; -89.379136, 32.218893; -86.870979, 31.788445; -88.138683, 34.627986; -87.938538, 36.596873; -88.043306, 35.94396

Novel plant genotypes

Authentication

ChIP-seq

Data deposition

[ ] Confirm that both raw and final processed data have been deposited in a public database such as GEO.

[ ] Confirm that you have deposited or provided access to graph files (e.g. BED files) for the called peaks.

Data access links
May remain private before publication.

Files in database submission

Genome browser session
(e.g. UCSC)

Methodology

Replicates

Sequencing depth

Antibodies

Peak calling parameters

Data quality

Software

Flow Cytometry

Plots

Confirm that:

- [ ] The axis labels state the marker and fluorochrome used (e.g. CD4-FITC).
- [ ] The axis scales are clearly visible. Include numbers along axes only for bottom left plot of group (a 'group' is an analysis of identical markers).
- [ ] All plots are contour plots with outliers or pseudocolor plots.
- [ ] A numerical value for number of cells or percentage (with statistics) is provided.

Methodology

Sample preparation

Instrument

Software

Cell population abundance

Gating strategy

- [ ] Tick this box to confirm that a figure exemplifying the gating strategy is provided in the Supplementary Information.

Magnetic resonance imaging

Experimental design

Design type

Design specifications

Behavioral performance measures

Imaging type(s)

Field strength

Sequence & imaging parameters

Area of acquisition

Diffusion MRI [ ] Used [ ] Not used

Preprocessing

Preprocessing software

Normalization

Normalization template

Noise and artifact removal

Volume censoring

Statistical modeling & inference

Model type and settings

Effect(s) tested

Specify type of analysis: [ ] Whole brain [ ] ROI-based [ ] Both

Statistic type for inference

(See Eklund et al. 2016)

Correction

Models & analysis

n/a | Involved in the study

- [ ] [ ] Functional and/or effective connectivity
- [ ] [ ] Graph analysis
- [ ] [ ] Multivariate modeling or predictive analysis

Functional and/or effective connectivity

Graph analysis

Multivariate modeling and predictive analysis

This checklist template is licensed under a Creative Commons Attribution 4.0 International License, which permits use, sharing, adaptation, distribution and reproduction in any medium or format, as long as you give appropriate credit to the original author(s) and the source, provide a link to the Creative Commons license, and indicate if changes were made. The images or other third party material in this article are included in the article's Creative Commons license, unless indicated otherwise in a credit line to the material. If material is not included in the article's Creative Commons license and your intended use is not permitted by statutory regulation or exceeds the permitted use, you will need to obtain permission directly from the copyright holder. To view a copy of this license, visit http://creativecommons.org/licenses/by/4.0/
