# Supplementary material for: A multidimensional selective landscape drives adaptive divergence between and within closely related Phlox species
Source: Nat Commun. 2024 May 31;15:4661. doi: 10.1038/s41467-024-49075-6 (PMC11143288; doi:10.1038/s41467-024-49075-6)
Supplement: Supplementary file 4 — Description of Additional Supplementary Files [file 41467_2024_49075_MOESM4_ESM.pdf]

Description of Additional Supplementary Files

File Name: Supplementary Data 1
Description: Supplementary Data 1 includes descriptions and source of environmental variables used in niche modeling.

File Name: Supplementary Data 2
Description: Supplementary Data 2 includes information about each source population used for niche modeling analysis.

File Name: Supplementary Data 3
Description: Supplementary Data 3 includes the geographic distance, genetic distance and environmental distance between the source population of each individual and the experimental garden in which that individual was grown.

File Name: Supplementary Data 4
Description: Supplementary Data 4 shows the results from each regression model testing the relationship between distance (geographic, genetic and environmental) and fitness for each species in each garden. Bold indicates significant model as determined by uncorrected $p < 0.05$.

File Name: Supplementary Data 5
Description: Supplementary Data 5 lists the source populations for the plants used in the experimental gardens with GPS points and number of individuals sourced.

File Name: Supplementary Data 6
Description: Supplementary Data 6 describes the location and habitat for each of the experimental garden sites.

File Name: Supplementary Data 7
Description: Supplementary Data 7 summarizes leaf trait PCA with proportion and cumulative variance explained by the first three PC axes.

File Name: Supplementary Data 8
Description: Supplementary Data 8 includes the results from the linear models about how fitness predicts the first two PCs of leaf trait variation.

File Name: Supplementary Data 9
Description: Supplementary Data 9 shows results from the linear models of leaf trait PC1 and PC2 predicting fitness within and across all three *Phlox* species.

File Name: Supplementary Data 10
Description: Supplementary Data 10 is a summary of all regression coefficients of fitness measures and the first three PCs of leaf traits for all three species and just for *Phlox pilosa*.

File Name: Supplementary Data 11
Description: Supplementary Data 11 is results from ANOVA model comparing leaf traits between field and greenhouse grown *Phlox* plants.
